# Supplementary material for: Associations of perceived neighborhood factors and Alzheimer’s disease polygenic score with cognition: Evidence from the Health and Retirement Study
Source: PLoS One. 2025 Nov 20;20(11):e0336403. doi: 10.1371/journal.pone.0336403 (PMC12633890; doi:10.1371/journal.pone.0336403)
Supplement: S1 Table — an (%); Mean (SD). bPearson’s Chi-squared test; Welch Two Sample t-test. (DOCX) [file pone.0336403.s001.docx]

**Supplemental Table 1.** Characteristics of Included and Excluded Participants with Neighborhood disadvantage index in the Health and Retirement Study (2008-2010 Waves).

|  | **Overall** | **Cognitive Impairment** | | | **CIND** | | | **Dementia** | | |  |
| --- | --- | --- | --- | --- | --- | --- | --- | --- | --- | --- | --- |
| **Main Variables** |  | **Excluded sample** | **Included sample** | **p-value**^b^ | **Excluded sample** | **Included sample** | **p-value**^b^ | **Excluded sample** | **Included sample** | **p-value**^b^ |  |
|  | **(N=14,562)**^a^ | **(N=7,736)** ^a^ | **(N=6,826)** ^a^ |  | **(N=7,816)** ^a^ | **(N=6,746)** ^a^ |  | **(N=6,802)** ^a^ | **(N=7,760)** ^a^ |  |  |
| **CIND & Dementia** |  |  |  | **<0.001** |  |  | **<0.001** |  |  | **<0.001** |  |
| Yes | 5,453 (41%) | 3,381 (53%) | 2,072 (30%) |  | 3,461 (54%) | 1,992 (30%) |  | 2,582 (48%) | 2,871 (37%) |  |  |
| No | 7,700 (59%) | 2,946 (47%) | 4,754 (70%) |  | 2,946 (46%) | 4,754 (70%) |  | 2,811 (52%) | 4,889 (63%) |  |  |
| Missing | 1,409 | 1,409 |  |  | 1,409 |  |  | 1,409 |  |  |  |
| **CIND** |  |  |  |  |  |  | **<0.001** |  |  | **<0.001** |  |
| Yes | 5,010 (38%) | 3,018 (48%) | 1,992 (29%) | **<0.001** | 3,018 (47%) | 1,992 (30%) |  | 2,332 (43%) | 2,678 (35%) |  |  |
| No | 8,143 (62%) | 3,309 (52%) | 4,834 (71%) |  | 3,389 (53%) | 4,754 (70%) |  | 3,061 (57%) | 5,082 (65%) |  |  |
| Missing | 1,409 | 1,409 |  |  | 1,409 |  |  | 1,409 |  |  |  |
| **Dementia** |  |  |  | **<0.001** |  |  | **<0.001** |  |  | **<0.001** |  |
| Yes | 1,414 (11%) | 1,125 (18%) | 289 (4.2%) |  | 1,205 (19%) | 209 (3.1%) |  | 796 (15%) | 618 (8.0%) |  |  |
| No | 11,739 (89%) | 5,202 (82%) | 6,537 (96%) |  | 5,202 (81%) | 6,537 (97%) |  | 4,597 (85%) | 7,142 (92%) |  |  |
| Missing | 1,409 | 1,409 |  |  | 1,409 |  |  | 1,409 |  |  |  |
| **Neighborhood disadvantage index** | 0.00 (1.00) | 0.11 (1.07) | -0.13 (0.90) | **<0.001** | 0.11 (1.07) | -0.13 (0.90) | **<0.001** | 0.11 (1.07) | -0.10 (0.93) | **<0.001** |  |
| **Neighborhood disadvantage index (Binary)** |  |  |  | **<0.001** |  |  | **<0.001** |  |  | **<0.001** |  |
| The least disadvantaged neighborhoods (<=0) | 8,917 (61%) | 4,380 (57%) | 4,537 (66%) |  | 4,429 (57%) | 4,488 (67%) |  | 3,847 (57%) | 5,070 (65%) |  |  |
| The most disadvantaged neighborhoods (>0) | 5,645 (39%) | 3,356 (43%) | 2,289 (34%) |  | 3,387 (43%) | 2,258 (33%) |  | 2,955 (43%) | 2,690 (35%) |  |  |
| **Baseline wave** |  |  |  | **<0.001** |  |  | **<0.001** |  |  | **<0.001** |  |
| Wave 1 (2008) | 6,714 (46%) | 3,706 (48%) | 3,008 (44%) |  | 3,743 (48%) | 2,971 (44%) |  | 3,278 (48%) | 3,436 (44%) |  |  |
| Wave 2 (2010) | 7,848 (54%) | 4,030 (52%) | 3,818 (56%) |  | 4,073 (52%) | 3,775 (56%) |  | 3,524 (52%) | 4,324 (56%) |  |  |
| **Polygenic score for AD** |  |  |  |  |  |  |  |  |  |  |  |
| European ancestry | -0.02 (1.00) | 0.14 (1.04) | -0.08 (0.98) | - | 0.14 (1.04) | -0.09 (0.98) | - | 0.20 (1.05) | -0.08 (0.98) | - |  |
| Missing | 5,685 | 4,982 | 703 |  | 4,990 | 695 |  | 4,714 | 971 |  |  |
| African ancestry | 0.02 (0.99) | -0.01 (1.03) | 0.06 (0.93) | - | -0.01 (1.03) | 0.06 (0.93) | - | -0.04 (1.11) | 0.06 (0.91) | - |  |
| Missing | 13,031 | 6908 | 6123 |  | 6,980 | 6,051 |  | 6,242 | 6,789 |  |  |
| **Polygenic score for AD (Binary)** |  |  |  |  |  |  |  |  |  |  |  |
| European ancestry |  |  |  | - |  |  | - |  |  | - |  |
| Below 75% | 6,657 (75%) | 1,888 (69%) | 4,769 (78%) |  | 1,938 (69%) | 4,719 (78%) |  | 1,390 (67%) | 5,267 (78%) |  |  |
| Above 75% | 2,220 (25%) | 866 (31%) | 1,354 (22%) |  | 888 (31%) | 1,332 (22%) |  | 698 (33%) | 1,522 (22%) |  |  |
| Missing | 5,685 | 4,982 | 703 |  | 4,990 | 695 |  | 4,714 | 971 |  |  |
| African ancestry |  |  |  | - |  |  | - |  |  | - |  |
| Below 75% | 1,148 (75%) | 614 (74%) | 534 (76%) |  | 617 (74%) | 531 (76%) |  | 407 (73%) | 741 (76%) |  |  |
| Above 75% | 383 (25%) | 214 (26%) | 169 (24%) |  | 219 (26%) | 164 (24%) |  | 153 (27%) | 230 (24%) |  |  |
| Missing | 13,031 | 6,908 | 6,123 |  | 6,980 | 6,051 |  | 6,242 | 6,789 |  |  |
| **APOE E4 status** |  |  |  | 0.706 |  |  | 0.623 |  |  | 0.185 |  |
| Any copies of e4 | 3,089 (27%) | 1,282 (27%) | 1,807 (26%) |  | 1,306 (27%) | 1,783 (26%) |  | 995 (26%) | 2,094 (27%) |  |  |
| No copies of e4 | 8,523 (73%) | 3,504 (73%) | 5,019 (74%) |  | 3,560 (73%) | 4,963 (74%) |  | 2,857 (74%) | 5,666 (73%) |  |  |
| Missing | 2,950 | 2,950 |  |  | 2,950 |  |  | 2,950 |  |  |  |
| **Sex** |  |  |  | **<0.001** |  |  | **0.018** |  |  | **<0.001** |  |
| Male | 6,011 (41%) | 3,337 (43%) | 2,674 (39%) |  | 3,370 (43%) | 2,641 (39%) |  | 2,918 (43%) | 3,093 (40%) |  |  |
| Female | 8,551 (59%) | 4,399 (57%) | 4,152 (61%) |  | 4,446 (57%) | 4,105 (61%) |  | 3,884 (57%) | 4,667 (60%) |  |  |
| **Age** | 67.57 (10.92) | 68.42 (11.56) | 66.61 (10.06) | **<0.001** | 68.46 (11.56) | 66.54 (10.04) | **<0.001** | 67.89 (11.61) | 67.29 (10.28) | **0.001** |  |
| **Education Level** |  |  |  | **<0.001** |  |  | **<0.001** |  |  | **<0.001** |  |
| > High School / GED | 4,128 (28%) | 1,766 (23%) | 2,362 (35%) |  | 1,792 (23%) | 2,336 (35%) |  | 1,639 (24%) | 2,489 (32%) |  |  |
| High School / GED | 7,895 (54%) | 3,974 (51%) | 3,921 (57%) |  | 4,019 (51%) | 3,876 (57%) |  | 3,470 (51%) | 4,425 (57%) |  |  |
| < High School / GED | 2,536 (17%) | 1,993 (26%) | 543 (8.0%) |  | 2,002 (26%) | 534 (7.9%) |  | 1,690 (25%) | 846 (11%) |  |  |
| Missing | 3 | 3 |  |  | 3 |  |  | 3 |  |  |  |
| **Poverty Status** |  |  |  | **<0.001** |  |  | **<0.001** |  |  | **<0.001** |  |
| Above Poverty threshold | 13,179 (91%) | 6,661 (87%) | 6,518 (95%) |  | 6,738 (87%) | 6,441 (95%) |  | 5,859 (88%) | 7,320 (94%) |  |  |
| Below Poverty threshold | 1,270 (8.8%) | 962 (13%) | 308 (4.5%) |  | 965 (13%) | 305 (4.5%) |  | 830 (12%) | 440 (5.7%) |  |  |
| Missing | 113 | 113 |  |  | 113 |  |  | 113 |  |  |  |
| **Social Ladder** | 6.41 (1.76) | 6.26 (1.85) | 6.55 (1.66) | **<0.001** | 6.27 (1.85) | 6.55 (1.66) | **<0.001** | 6.27 (1.85) | 6.51 (1.68) | **<0.001** |  |
| Missing | 1,367 | 1,367 |  |  | 1,367 |  |  | 1,367 |  |  |  |
| **Sensitivity Variables** | **Overall** | **Excluded sample** | **Included sample** | **p-value** | **Excluded sample** | **Included sample** | **p-value** | **Excluded sample** | **Included sample** | **p-value** |  |
| **Smoking status** |  |  |  | **<0.001** |  |  | **<0.001** |  |  | **<0.001** |  |
| Current Smoker | 1,968 (14%) | 1,149 (15%) | 819 (12%) |  | 1,155 (15%) | 813 (12%) |  | 1,022 (15%) | 946 (12%) |  |  |
| Former Smoker | 6,188 (43%) | 3,271 (43%) | 2,917 (43%) |  | 3,311 (43%) | 2,877 (43%) |  | 2,830 (42%) | 3,358 (44%) |  |  |
| Never Smoke | 6,307 (44%) | 3,257 (42%) | 3,050 (45%) |  | 3,289 (42%) | 3,018 (45%) |  | 2,896 (43%) | 3,411 (44%) |  |  |
| Missing | 99 | 59 | 40 |  | 61 | 38 |  | 54 | 45 |  |  |
| **Drinking (# drinks/day when drinks)** | 0.76 (1.47) | 0.72 (1.55) | 0.81 (1.37) | **<0.001** | 0.72 (1.55) | 0.81 (1.37) | **<0.001** | 0.73 (1.56) | 0.78 (1.38) | **0.026** |  |
| Missing | 14 | 10 | 4 |  | 10 | 4 |  | 9 | 5 |  |  |
| **BMI** | 28.43 (6.02) | 28.22 (6.02) | 28.67 (6.00) | **<0.001** | 28.21 (6.02) | 28.68 (6.00) | **<0.001** | 28.23 (5.99) | 28.60 (6.04) | **<0.001** |  |
| Missing | 178 | 127 | 51 |  | 128 | 50 |  | 117 | 61 |  |  |
| **Ever have Diabetes** |  |  |  | **<0.001** |  |  |  |  |  | **<0.001** |  |
| Yes | 2,979 (20%) | 1,850 (24%) | 1,129 (17%) |  | 1,860 (24%) | 1,119 (17%) |  | 1,610 (24%) | 1,369 (18%) |  |  |
| No | 11,583 (80%) | 5,886 (76%) | 5,697 (83%) |  | 5,956 (76%) | 5,627 (83%) |  | 5,192 (76%) | 6,391 (82%) |  |  |
| **Eyesight** |  |  |  | **<0.001** |  |  | **<0.001** |  |  | **<0.001** |  |
| Excellent | 1,419 (9.8%) | 690 (8.9%) | 729 (11%) |  | 698 (8.9%) | 721 (11%) |  | 626 (9.2%) | 793 (10%) |  |  |
| Very good | 3,956 (27%) | 1,742 (23%) | 2,214 (32%) |  | 1,765 (23%) | 2,191 (32%) |  | 1,550 (23%) | 2,406 (31%) |  |  |
| Good | 6,136 (42%) | 3,250 (42%) | 2,886 (42%) |  | 3,283 (42%) | 2,853 (42%) |  | 2,841 (42%) | 3,295 (42%) |  |  |
| Fair | 2,278 (16%) | 1,485 (19%) | 793 (12%) |  | 1,497 (19%) | 781 (12%) |  | 1,295 (19%) | 983 (13%) |  |  |
| Poor | 711 (4.9%) | 518 (6.7%) | 193 (2.8%) |  | 521 (6.7%) | 190 (2.8%) |  | 443 (6.5%) | 268 (3.5%) |  |  |
| Blind | 42 (0.3%) | 34 (0.4%) | 8 (0.1%) |  | 35 (0.4%) | 7 (0.1%) |  | 30 (0.4%) | 12 (0.2%) |  |  |
| Unknown | 20 | 17 | 3 |  | 17 | 3 |  | 17 | 3 |  |  |
| **Hearing** |  |  |  | **<0.001** |  |  | **<0.001** |  |  | **<0.001** |  |
| Excellent | 2,638 (18%) | 1,360 (18%) | 1,278 (19%) |  | 1,369 (18%) | 1,269 (19%) |  | 1,219 (18%) | 1,419 (18%) |  |  |
| Very good | 3,998 (27%) | 1,871 (24%) | 2,127 (31%) |  | 1,893 (24%) | 2,105 (31%) |  | 1,683 (25%) | 2,315 (30%) |  |  |
| Good | 4,952 (34%) | 2,701 (35%) | 2,251 (33%) |  | 2,729 (35%) | 2,223 (33%) |  | 2,356 (35%) | 2,596 (33%) |  |  |
| Fair | 2,212 (15%) | 1,301 (17%) | 911 (13%) |  | 1,320 (17%) | 892 (13%) |  | 1,114 (16%) | 1,098 (14%) |  |  |
| Poor | 757 (5.2%) | 500 (6.5%) | 257 (3.8%) |  | 502 (6.4%) | 255 (3.8%) |  | 427 (6.3%) | 330 (4.3%) |  |  |
| Unknown | 5 | 3 | 2 |  | 3 | 2 |  | 3 | 2 |  |  |
| **Brain Condition** |  |  |  | **<0.001** |  |  | **<0.001** |  |  | **<0.001** |  |
| Yes | 3,157 (22%) | 1,847 (24%) | 1,310 (19%) |  | 1,867 (24%) | 1,290 (19%) |  | 1,608 (24%) | 1,549 (20%) |  |  |
| No | 11,405 (78%) | 5,889 (76%) | 5,516 (81%) |  | 5,949 (76%) | 5,456 (81%) |  | 5,194 (76%) | 6,211 (80%) |  |  |
| **Chronic Condition** |  |  |  | **<0.001** |  |  | **<0.001** |  |  | **<0.001** |  |
| None | 2,387 (16%) | 1,193 (15%) | 1,194 (17%) |  | 1,206 (15%) | 1,181 (18%) |  | 1,084 (16%) | 1,303 (17%) |  |  |
| 1-2 | 7,942 (55%) | 4,043 (52%) | 3,899 (57%) |  | 4,084 (52%) | 3,858 (57%) |  | 3,593 (53%) | 4,349 (56%) |  |  |
| >= 3 | 4,233 (29%) | 2,500 (32%) | 1,733 (25%) |  | 2,526 (32%) | 1,707 (25%) |  | 2,125 (31%) | 2,108 (27%) |  |  |
| **Depression** | 1.38 (1.93) | 1.66 (2.09) | 1.08 (1.71) | **<0.001** | 1.66 (2.08) | 1.08 (1.71) | **<0.001** | 1.38 (1.93) | 1.66 (2.09) | **<0.001** |  |
| Missing | 415 | 415 |  |  | 415 |  |  | 415 |  |  |  |
| ^a^n (%); Mean (SD) | | | | | | | | | | |  |
| ^b^Pearson's Chi-squared test; Welch Two Sample t-test  Brian Condition includes stroke, psychiatric problems; Chronic Condition includes high blood pressure, diabetes, cancer, lung disease, heart disease, and arthritis | | | | | | | | | | |  |
