# Supplementary material for: Associations of perceived neighborhood factors and Alzheimer’s disease polygenic score with cognition: Evidence from the Health and Retirement Study
Source: PLoS One. 2025 Nov 20;20(11):e0336403. doi: 10.1371/journal.pone.0336403 (PMC12633890; doi:10.1371/journal.pone.0336403)
Supplement: S2 Table — an (%); Mean (SD). (DOCX) [file pone.0336403.s002.docx]

**Supplemental Table 2**: Sample distribution by genetic ancestry in the Health and Retirement Study, wave 2008-2010.

|  | **Cognitive Impairment** | | | **CIND** | | | **Dementia** | | |
| --- | --- | --- | --- | --- | --- | --- | --- | --- | --- |
| **Main Variables** | **Study sample** | **African ancestry** | **European ancestry** | **Study sample** | **African ancestry** | **European ancestry** | **Study sample** | **African ancestry** | **European ancestry** |
|  | **(N=6,826)** ^a^ | **(N=703)** ^a^ | **(N=6,123)** ^a^ | **(N=6,746)** ^a^ | **(N=695)** ^a^ | **(N=6,051)** ^a^ | **(N=7,760)** ^a^ | **(N=971)** ^a^ | **(N=6,789)** ^a^ |
| **Neighborhood disadvantage index** | -0.13 (0.90) | 0.47 (1.04) | -0.20 (0.85) | -0.13 (0.90) | 0.47 (1.04) | -0.20 (0.85) | -0.10 (0.93) | 0.49 (1.06) | -0.18 (0.87) |
| **Neighborhood disadvantage index (Binary)** |  |  |  |  |  |  |  |  |  |
| The least disadvantaged neighborhoods (<=0) | 4,537 (66%) | 274 (39%) | 4,263 (70%) | 4,488 (67%) | 272 (39%) | 4,216 (70%) | 5,070 (65%) | 372 (38%) | 4,698 (69%) |
| The most disadvantaged neighborhoods (>0) | 2,289 (34%) | 429 (61%) | 1,860 (30%) | 2,258 (33%) | 423 (61%) | 1,835 (30%) | 2,690 (35%) | 599 (62%) | 2,091 (31%) |
| **PGS-AD** |  |  |  |  |  |  |  |  |  |
| European ancestry | -0.08 (0.98) | - | -0.08 (0.98) | -0.09 (0.98) | - | -0.09 (0.98) | -0.08 (0.98) | - | -0.08 (0.98) |
| African ancestry | 0.06 (0.93) | 0.06 (0.93) | - | 0.06 (0.93) | 0.06 (0.93) | - | 0.06 (0.91) | 0.06 (0.91) | - |
| **PGS-AD (Binary)** |  |  |  |  |  |  |  |  |  |
| European ancestry |  |  |  |  |  |  |  |  |  |
| Below 75% | 4,769 (78%) | - | 4,769 (78%) | 4,719 (78%) | - | 4,719 (78%) | 5,267 (78%) | - | 5,267 (78%) |
| Above 75% | 1,354 (22%) | - | 1,354 (22%) | 1,332 (22%) | - | 1,332 (22%) | 1,522 (22%) | - | 1,522 (22%) |
| African ancestry |  |  |  |  |  |  |  |  |  |
| Below 75% | 534 (76%) | 534 (76%) | - | 531 (76%) | 531 (76%) | - | 741 (76%) | 741 (76%) | - |
| Above 75% | 169 (24%) | 169 (24%) | - | 164 (24%) | 164 (24%) | - | 230 (24%) | 230 (24%) | - |
| **APOE E4 status** |  |  |  |  |  |  |  |  |  |
| Any copies of e4 | 1,807 (26%) | 261 (37%) | 1,546 (25%) | 1,783 (26%) | 257 (37%) | 1,526 (25%) | 2,094 (27%) | 356 (37%) | 1,738 (26%) |
| No copies of e4 | 5,019 (74%) | 442 (63%) | 4,577 (75%) | 4,963 (74%) | 438 (63%) | 4,525 (75%) | 5,666 (73%) | 615 (63%) | 5,051 (74%) |
| **Sex** |  |  |  |  |  |  |  |  |  |
| Female | 4,152 (61%) | 491 (70%) | 3,661 (60%) | 4,105 (61%) | 485 (70%) | 3,620 (60%) | 4,667 (60%) | 653 (67%) | 4,014 (59%) |
| Male | 2,674 (39%) | 212 (30%) | 2,462 (40%) | 2,641 (39%) | 210 (30%) | 2,431 (40%) | 3,093 (40%) | 318 (33%) | 2,775 (41%) |
| **Age** | 66.61 (10.06) | 62.06 (8.84) | 67.14 (10.06) | 66.54 (10.04) | 62.08 (8.86) | 67.05 (10.04) | 67.29 (10.28) | 63.67 (9.56) | 67.81 (10.27) |
| **Education Level** |  |  |  |  |  |  |  |  |  |
| > High School / GED | 2,362 (35%) | 195 (28%) | 2,167 (35%) | 2,336 (35%) | 192 (28%) | 2,144 (35%) | 2,489 (32%) | 210 (22%) | 2,279 (34%) |
| High School / GED | 3,921 (57%) | 393 (56%) | 3,528 (58%) | 3,876 (57%) | 388 (56%) | 3,488 (58%) | 4,425 (57%) | 528 (54%) | 3,897 (57%) |
| < High School / GED | 543 (8.0%) | 115 (16%) | 428 (7.0%) | 534 (7.9%) | 115 (17%) | 419 (6.9%) | 846 (11%) | 233 (24%) | 613 (9.0%) |
| **Poverty Status** |  |  |  |  |  |  |  |  |  |
| Above Poverty threshold | 6,518 (95%) | 594 (84%) | 5,924 (97%) | 6,441 (95%) | 587 (84%) | 5,854 (97%) | 7,320 (94%) | 783 (81%) | 6,537 (96%) |
| Below Poverty threshold | 308 (4.5%) | 109 (16%) | 199 (3.3%) | 305 (4.5%) | 108 (16%) | 197 (3.3%) | 440 (5.7%) | 188 (19%) | 252 (3.7%) |
| **Baseline wave** |  |  |  |  |  |  |  |  |  |
| Wave 1 (2008) | 3,008 (44%) | 273 (39%) | 2,735 (45%) | 2,971 (44%) | 271 (39%) | 2,700 (45%) | 3,436 (44%) | 391 (40%) | 3,045 (45%) |
| Wave 2 (2010) | 3,818 (56%) | 430 (61%) | 3,388 (55%) | 3,775 (56%) | 424 (61%) | 3,351 (55%) | 4,324 (56%) | 580 (60%) | 3,744 (55%) |
| **Social Ladder** | -0.08 (0.94) | 0.24 (1.02) | -0.11 (0.93) | 6.55 (1.66) | 5.99 (1.80) | 6.61 (1.63) | 6.51 (1.68) | 5.95 (1.83) | 6.59 (1.65) |
| **Sensitivity Variables** | **Sensitivity analytic sample** | **African ancestry** | **European ancestry** | **Sensitivity analytic sample** | **African ancestry** | **European ancestry** | **Sensitivity analytic sample** | **African ancestry** | **European ancestry** |
| **Smoking status** |  |  |  |  |  |  |  |  |  |
| Current Smoker | 819 (12%) | 139 (20%) | 680 (11%) | 813 (12%) | 138 (20%) | 675 (11%) | 946 (12%) | 186 (19%) | 760 (11%) |
| Former Smoker | 2,917 (43%) | 266 (38%) | 2,651 (44%) | 2,877 (43%) | 262 (38%) | 2,615 (43%) | 3,358 (44%) | 386 (40%) | 2,972 (44%) |
| Never Smoke | 3,050 (45%) | 296 (42%) | 2,754 (45%) | 3,018 (45%) | 293 (42%) | 2,725 (45%) | 3,411 (44%) | 395 (41%) | 3,016 (45%) |
| **BMI** | 28.67 (6.00) | 31.13 (7.28) | 28.38 (5.77) | 28.68 (6.00) | 31.14 (7.28) | 28.40 (5.77) | 28.60 (6.04) | 30.86 (7.14) | 28.28 (5.79) |
| **Drinking (# drinks/day when drinks)** | 0.81 (1.37) | 0.66 (1.31) | 0.82 (1.37) | 0.81 (1.37) | 0.66 (1.32) | 0.83 (1.38) | 0.78 (1.38) | 0.64 (1.33) | 0.81 (1.39) |
| **Ever have Diabetes** |  |  |  |  |  |  |  |  |  |
| Yes | 1,129 (17%) | 168 (24%) | 961 (16%) | 1,119 (17%) | 166 (24%) | 953 (16%) | 1,369 (18%) | 264 (27%) | 1,105 (16%) |
| No | 5,697 (83%) | 535 (76%) | 5,162 (84%) | 5,627 (83%) | 529 (76%) | 5,098 (84%) | 6,391 (82%) | 707 (73%) | 5,684 (84%) |
| **Eyesight** |  |  |  |  |  |  |  |  |  |
| Excellent | 729 (11%) | 48 (6.8%) | 681 (11%) | 721 (11%) | 48 (6.9%) | 673 (11%) | 793 (10%) | 55 (5.7%) | 738 (11%) |
| Very good | 2,214 (32%) | 140 (20%) | 2,074 (34%) | 2,191 (32%) | 137 (20%) | 2,054 (34%) | 2,406 (31%) | 181 (19%) | 2,225 (33%) |
| Good | 2,886 (42%) | 340 (48%) | 2,546 (42%) | 2,853 (42%) | 337 (48%) | 2,516 (42%) | 3,295 (42%) | 454 (47%) | 2,841 (42%) |
| Fair | 793 (12%) | 133 (19%) | 660 (11%) | 781 (12%) | 132 (19%) | 649 (11%) | 983 (13%) | 211 (22%) | 772 (11%) |
| Poor | 193 (2.8%) | 42 (6.0%) | 151 (2.5%) | 190 (2.8%) | 41 (5.9%) | 149 (2.5%) | 268 (3.5%) | 70 (7.2%) | 198 (2.9%) |
| Blind | 8 (0.1%) | 0 (0%) | 8 (0.1%) | 7 (0.1%) | 0 (0%) | 7 (0.1%) | 12 (0.2%) | 0 (0%) | 12 (0.2%) |
| **Hearing** |  |  |  |  |  |  |  |  |  |
| Excellent | 1,278 (19%) | 162 (23%) | 1,116 (18%) | 1,269 (19%) | 162 (23%) | 1,107 (18%) | 1,419 (18%) | 207 (21%) | 1,212 (18%) |
| Very good | 2,127 (31%) | 203 (29%) | 1,924 (31%) | 2,105 (31%) | 200 (29%) | 1,905 (31%) | 2,315 (30%) | 260 (27%) | 2,055 (30%) |
| Good | 2,251 (33%) | 258 (37%) | 1,993 (33%) | 2,223 (33%) | 254 (37%) | 1,969 (33%) | 2,596 (33%) | 376 (39%) | 2,220 (33%) |
| Fair | 911 (13%) | 66 (9.4%) | 845 (14%) | 892 (13%) | 65 (9.4%) | 827 (14%) | 1,098 (14%) | 110 (11%) | 988 (15%) |
| Poor | 257 (3.8%) | 14 (2.0%) | 243 (4.0%) | 255 (3.8%) | 14 (2.0%) | 241 (4.0%) | 330 (4.3%) | 18 (1.9%) | 312 (4.6%) |
| **Brain Condition** |  |  |  |  |  |  |  |  |  |
| Yes | 1,310 (19%) | 122 (17%) | 1,188 (19%) | 1,290 (19%) | 120 (17%) | 1,170 (19%) | 1,549 (20%) | 173 (18%) | 1,376 (20%) |
| No | 5,516 (81%) | 581 (83%) | 4,935 (81%) | 5,456 (81%) | 575 (83%) | 4,881 (81%) | 6,211 (80%) | 798 (82%) | 5,413 (80%) |
| **Chronic Condition** |  |  |  |  |  |  |  |  |  |
| None | 1,194 (17%) | 116 (17%) | 1,078 (18%) | 1,181 (18%) | 115 (17%) | 1,066 (18%) | 1,303 (17%) | 153 (16%) | 1,150 (17%) |
| 1-2 | 3,899 (57%) | 392 (56%) | 3,507 (57%) | 3,858 (57%) | 387 (56%) | 3,471 (57%) | 4,349 (56%) | 508 (52%) | 3,841 (57%) |
| >= 3 | 1,733 (25%) | 195 (28%) | 1,538 (2 | 1,707 (25%) | 193 (28%) | 1,514 (25%) | 2,108 (27%) | 310 (32%) | 1,798 (26%) |
| **Depression** | 1.08 (1.71) | 1.47 (1.90) | 1.03 (1.68) | 1.08 (1.71) | 1.47 (1.90) | 1.03 (1.68) | 1.15 (1.76) | 1.62 (2.00) | 1.09 (1.72) |

^a^n (%); Mean (SD)

Brian Condition includes stroke, psychiatric problems; Chronic Condition includes high blood pressure, diabetes, cancer, lung disease, heart disease, and arthritis
