# Supplementary material for: Associations of perceived neighborhood factors and Alzheimer’s disease polygenic score with cognition: Evidence from the Health and Retirement Study
Source: PLoS One. 2025 Nov 20;20(11):e0336403. doi: 10.1371/journal.pone.0336403 (PMC12633890; doi:10.1371/journal.pone.0336403)
Supplement: S3 Table — (DOCX) [file pone.0336403.s003.docx]

**Supplemental Table 3:** Hazard Ratios from survival analysis with binary Neighborhood disadvantage index stratified by Ancestry, estimates present the association for the most Disadvantage Neighborhoods with incident cognitive impairment (CIND and dementia), CIND and dementia, relative to normal cognition and non-dementia respectively in the US Health and Retirement Study (2008-2010 Waves).

|  | **Cognitive Impairment vs. Normal Cognition, European Ancestry (n=6,123)** | | | | | | | | | **CIND vs. Normal Cognition, European Ancestry (n=6,051)** | | | | | | | | | **Dementia vs. Non-dementia, European Ancestry (n=6,789)** | | | | | | | | |
| --- | --- | --- | --- | --- | --- | --- | --- | --- | --- | --- | --- | --- | --- | --- | --- | --- | --- | --- | --- | --- | --- | --- | --- | --- | --- | --- | --- |
|  | **Model 1** | | | **Model 2** | | | **Model 3** | | | **Model 1** | | | **Model 2** | | | **Model 3** | | | **Model 1** | | | **Model 2** | | | **Model 3** | | |
|  | **HR** | **95% CI** | **p-value** | **HR** | **95% CI** | **p-value** | **HR** | **95% CI** | **p-value** | **HR** | **95% CI** | **p-value** | **HR** | **95% CI** | **p-value** | **HR** | **95% CI** | **p-value** | **HR** | **95% CI** | **p-value** | **HR** | **95% CI** | **p-value** | **HR** | **95% CI** | **p-value** |
| **Neighborhood disadvantage index** |  |  |  |  |  |  |  |  |  |  |  |  |  |  |  |  |  |  |  |  |  |  |  |  |  |  |  |
| The least disadvantaged neighborhoods (<=0) | Ref | Ref | Ref | Ref | Ref | Ref | Ref | Ref | Ref | Ref | Ref | Ref | Ref | Ref | Ref | Ref | Ref | Ref | Ref | Ref | Ref | Ref | Ref | Ref | Ref | Ref | Ref |
| The most disadvantaged neighborhoods (>0) | 1.19 | 1.08, 1.32 | **<0.001** | 1.20 | 1.08, 1.33 | **<0.001** | 1.21 | 1.08, 1.36 | **0.001** | 1.18 | 1.06, 1.31 | **0.002** | 1.19 | 1.07, 1.31 | **0.001** | 1.20 | 1.06, 1.35 | **0.003** | 1.34 | 1.10, 1.63 | **0.004** | 1.33 | 1.09, 1.62 | **0.005** | 1.34 | 1.07, 1.68 | **0.011** |
| **Age** | 1.08 | 1.07, 1.08 | **<0.001** | 1.08 | 1.07, 1.08 | **<0.001** | 1.08 | 1.07, 1.08 | **<0.001** | 1.08 | 1.07, 1.08 | **<0.001** | 1.08 | 1.07, 1.08 | **<0.001** | 1.08 | 1.07, 1.08 | **<0.001** | 1.12 | 1.10, 1.13 | **<0.001** | 1.12 | 1.10, 1.13 | **<0.001** | 1.12 | 1.10, 1.13 | **<0.001** |
| **Sex** |  |  |  |  |  |  |  |  |  |  |  |  |  |  |  |  |  |  |  |  |  |  |  |  |  |  |  |
| Female | Ref | Ref | Ref | Ref | Ref | Ref | Ref | Ref | Ref | Ref | Ref | Ref | Ref | Ref | Ref | Ref | Ref | Ref | Ref | Ref | Ref | Ref | Ref | Ref | Ref | Ref | Ref |
| Male | 1.31 | 1.19, 1.44 | **<0.001** | 1.30 | 1.18, 1.43 | **<0.001** | 1.30 | 1.18, 1.43 | **<0.001** | 1.32 | 1.19, 1.45 | **<0.001** | 1.31 | 1.19, 1.45 | **<0.001** | 1.31 | 1.19, 1.45 | **<0.001** | 1.06 | 0.87, 1.28 | 0.600 | 1.06 | 0.88, 1.29 | 0.500 | 1.06 | 0.87, 1.29 | 0.500 |
| **Education** |  |  |  |  |  |  |  |  |  |  |  |  |  |  |  |  |  |  |  |  |  |  |  |  |  |  |  |
| Above High School/GED | Ref | Ref | Ref | Ref | Ref | Ref | Ref | Ref | Ref | Ref | Ref | Ref | Ref | Ref | Ref | Ref | Ref | Ref | Ref | Ref | Ref | Ref | Ref | Ref | Ref | Ref | Ref |
| High School/GED | 1.62 | 1.44, 1.82 | **<0.001** | 1.62 | 1.44, 1.82 | **<0.001** | 1.62 | 1.44, 1.82 | **<0.001** | 1.65 | 1.46, 1.86 | **<0.001** | 1.65 | 1.47, 1.86 | **<0.001** | 1.65 | 1.47, 1.86 | **<0.001** | 1.59 | 1.23, 2.05 | **<0.001** | 1.61 | 1.24, 2.07 | **<0.001** | 1.61 | 1.24, 2.07 | **<0.001** |
| Less than High School/GED | 3.04 | 2.57, 3.60 | **<0.001** | 3.04 | 2.57, 3.59 | **<0.001** | 3.04 | 2.57, 3.59 | **<0.001** | 3.09 | 2.61, 3.67 | **<0.001** | 3.10 | 2.61, 3.67 | **<0.001** | 3.09 | 2.61, 3.67 | **<0.001** | 3.99 | 2.96, 5.37 | **<0.001** | 4.04 | 3.00, 5.44 | **<0.001** | 4.04 | 3.00, 5.44 | **<0.001** |
| **Poverty Status (Below)** |  |  |  |  |  |  |  |  |  |  |  |  |  |  |  |  |  |  |  |  |  |  |  |  |  |  |  |
| Above Poverty threshold | Ref | Ref | Ref | Ref | Ref | Ref | Ref | Ref | Ref | Ref | Ref | Ref | Ref | Ref | Ref | Ref | Ref | Ref | Ref | Ref | Ref | Ref | Ref | Ref | Ref | Ref | Ref |
| Below Poverty threshold | 1.28 | 0.99, 1.65 | 0.056 | 1.27 | 0.99, 1.64 | 0.063 | 1.27 | 0.99, 1.64 | 0.063 | 1.28 | 0.99, 1.66 | 0.060 | 1.27 | 0.98, 1.65 | 0.069 | 1.27 | 0.98, 1.65 | 0.069 | 2.04 | 1.39, 2.99 | **<0.001** | 2.07 | 1.41, 3.04 | **<0.001** | 2.07 | 1.41, 3.04 | **<0.001** |
| **APOE E4 status** |  |  |  |  |  |  |  |  |  |  |  |  |  |  |  |  |  |  |  |  |  |  |  |  |  |  |  |
| No copies of e4 | Ref | Ref | Ref | Ref | Ref | Ref | Ref | Ref | Ref | Ref | Ref | Ref | Ref | Ref | Ref | Ref | Ref | Ref | Ref | Ref | Ref | Ref | Ref | Ref | Ref | Ref | Ref |
| Any copies of e4 | 1.43 | 1.29, 1.59 | **<0.001** | 1.43 | 1.29, 1.59 | **<0.001** | 1.43 | 1.28, 1.58 | **<0.001** | 1.44 | 1.29, 1.60 | **<0.001** | 1.43 | 1.28, 1.59 | **<0.001** | 1.43 | 1.28, 1.59 | **<0.001** | 2.06 | 1.70, 2.50 | **<0.001** | 2.05 | 1.69, 2.49 | **<0.001** | 2.05 | 1.69, 2.49 | **<0.001** |
| **Social Ladder** | 0.93 | 0.90, 0.95 | **<0.001** | 0.93 | 0.90, 0.95 | **<0.001** | 0.93 | 0.90, 0.95 | **<0.001** | 0.92 | 0.90, 0.95 | **<0.001** | 0.92 | 0.89, 0.95 | **<0.001** | 0.92 | 0.89, 0.95 | **<0.001** | 0.98 | 0.93, 1.05 | 0.600 | 0.99 | 0.93, 1.05 | 0.700 | 0.99 | 0.93, 1.05 | 0.700 |
| **Baseline wave** |  |  |  |  |  |  |  |  |  |  |  |  |  |  |  |  |  |  |  |  |  |  |  |  |  |  |  |
| Wave 1 (2008) | Ref | Ref | Ref | Ref | Ref | Ref | Ref | Ref | Ref | Ref | Ref | Ref | Ref | Ref | Ref | Ref | Ref | Ref | Ref | Ref | Ref | Ref | Ref | Ref | Ref | Ref | Ref |
| Wave 2 (2010) | 0.86 | 0.78, 0.94 | **0.001** | 0.86 | 0.78, 0.95 | **0.002** | 0.86 | 0.78, 0.94 | **0.002** | 0.86 | 0.78, 0.95 | **0.002** | 0.86 | 0.78, 0.95 | **0.002** | 0.86 | 0.78, 0.95 | **0.002** | 0.81 | 0.67, 0.97 | **0.023** | 0.8 | 0.67, 0.97 | **0.020** | 0.8 | 0.67, 0.97 | **0.020** |
| **PGS-AD** |  |  |  |  |  |  |  |  |  |  |  |  |  |  |  |  |  |  |  |  |  |  |  |  |  |  |  |
| Below 75% | - | - | - | Ref | Ref | Ref | Ref | Ref | Ref | - | - | - | Ref | Ref | Ref | Ref | Ref | Ref | - | - | - | Ref | Ref | Ref | Ref | Ref | Ref |
| Above 75% | - | - | - | 1.12 | 1.01, 1.25 | **0.040** | 1.14 | 0.99, 1.30 | 0.061 | - | - | - | 1.12 | 1.00, 1.25 | 0.055 | 1.13 | 0.99, 1.30 | 0.078 | - | - | - | 1.19 | 0.96, 1.47 | 0.120 | 1.20 | 0.92, 1.57 | 0.200 |
| **Neighborhood* PGS-AD** | - | - | - | - | - | - | 0.96 | 0.76, 1.22 | 0.700 | - | - | - | - | - | - | 0.96 | 0.76, 1.22 | 0.700 | - | - | - | - | - | - | 0.96 | 0.62, 1.51 | 0.900 |
| RERI: The most disadvantaged neighborhoods*PGS-AD Above 75% | - | - | - | - | - | - | -0.02 | -0.31, 0.27 |  | - | - | - | - | - | - | -0.03 | -0.32, 0.27 |  | - | - | - | - | - | - | 0.01 | -0.61, 0.63 |  |
|  | **Cognitive Impairment vs. Normal Cognition, African Ancestry (n=703)** | | | | | | | | | **CIND vs. Normal Cognition, African Ancestry (n=695)** | | | | | | | | | **Dementia vs. Non-dementia, African Ancestry (n=971)** | | | | | | | | |
|  | **Model 1** | | | **Model 2** | | | **Model 3** | | | **Model 1** | | | **Model 2** | | | **Model 3** | | | **Model 1** | | | **Model 2** | | | **Model 3** | | |
|  | **HR** | **95% CI** | **p-value** | **HR** | **95% CI** | **p-value** | **HR** | **95% CI** | **p-value** | **HR** | **95% CI** | **p-value** | **HR** | **95% CI** | **p-value** | **HR** | **95% CI** | **p-value** | **HR** | **95% CI** | **p-value** | **HR** | **95% CI** | **p-value** | **HR** | **95% CI** | **p-value** |
| **Neighborhood disadvantage index** |  |  |  |  |  |  |  |  |  |  |  |  |  |  |  |  |  |  |  |  |  |  |  |  |  |  |  |
| The least disadvantaged neighborhoods (<=0) | Ref | Ref | Ref | Ref | Ref | Ref | Ref | Ref | Ref | Ref | Ref | Ref | Ref | Ref | Ref | Ref | Ref | Ref | Ref | Ref | Ref | Ref | Ref | Ref | Ref | Ref | Ref |
| The most disadvantaged neighborhoods (>0) | 1.05 | 0.83, 1.33 | 0.700 | 1.02 | 0.81, 1.30 | 0.800 | 1.13 | 0.86, 1.50 | 0.4 | 1.05 | 0.83, 1.33 | 0.700 | 1.03 | 0.81, 1.30 | 0.800 | 1.11 | 0.84, 1.48 | 0.5 | 1.17 | 0.85, 1.61 | 0.300 | 1.17 | 0.85, 1.61 | 0.300 | 1.21 | 0.84, 1.74 | 0.3 |
| **Age** | 1.05 | 1.04, 1.07 | **<0.001** | 1.05 | 1.04, 1.07 | **<0.001** | 1.05 | 1.04, 1.07 | **<0.001** | 1.05 | 1.04, 1.07 | **<0.001** | 1.05 | 1.04, 1.07 | **<0.001** | 1.05 | 1.04, 1.07 | **<0.001** | 1.09 | 1.07, 1.11 | **<0.001** | 1.09 | 1.07, 1.11 | **<0.001** | 1.09 | 1.07, 1.11 | **<0.001** |
| **Sex** |  |  |  |  |  |  |  |  |  |  |  |  |  |  |  |  |  |  |  |  |  |  |  |  |  |  |  |
| Female | Ref | Ref | Ref | Ref | Ref | Ref | Ref | Ref | Ref | Ref | Ref | Ref | Ref | Ref | Ref | Ref | Ref | Ref | Ref | Ref | Ref | Ref | Ref | Ref | Ref | Ref | Ref |
| Male | 1.19 | 0.93, 1.52 | 0.200 | 1.21 | 0.94, 1.54 | 0.130 | 1.21 | 0.94, 1.55 | 0.13 | 1.19 | 0.93, 1.52 | 0.200 | 1.19 | 0.93, 1.53 | 0.200 | 1.19 | 0.93, 1.53 | 0.2 | 1.32 | 0.96, 1.81 | 0.089 | 1.36 | 0.99, 1.88 | 0.059 | 1.36 | 0.99, 1.87 | 0.06 |
| **Education** |  |  |  |  |  |  |  |  |  |  |  |  |  |  |  |  |  |  |  |  |  |  |  |  |  |  |  |
| Above High School/GED | Ref | Ref | Ref | Ref | Ref | Ref | Ref | Ref | Ref | Ref | Ref | Ref | Ref | Ref | Ref | Ref | Ref | Ref | Ref | Ref | Ref | Ref | Ref | Ref | Ref | Ref | Ref |
| High School/GED | 1.51 | 1.12, 2.05 | **0.008** | 1.51 | 1.11, 2.04 | **0.009** | 1.5 | 1.11, 2.04 | **0.009** | 1.54 | 1.13, 2.10 | **0.006** | 1.54 | 1.12, 2.10 | **0.007** | 1.53 | 1.12, 2.10 | **0.007** | 2.14 | 1.16, 3.95 | **0.016** | 2.19 | 1.18, 4.05 | **0.013** | 2.18 | 1.18, 4.03 | **0.013** |
| Less than High School/GED | 3.04 | 2.13, 4.33 | **<0.001** | 2.96 | 2.07, 4.23 | **<0.001** | 2.99 | 2.09, 4.27 | **<0.001** | 3.12 | 2.18, 4.48 | **<0.001** | 3.02 | 2.10, 4.33 | **<0.001** | 3.03 | 2.11, 4.35 | **<0.001** | 5.51 | 2.96, 10.3 | **<0.001** | 5.63 | 3.01, 10.5 | **<0.001** | 5.63 | 3.01, 10.5 | **<0.001** |
| **Poverty Status** |  |  |  |  |  |  |  |  |  |  |  |  |  |  |  |  |  |  |  |  |  |  |  |  |  |  |  |
| Above Poverty threshold | Ref | Ref | Ref | Ref | Ref | Ref | Ref | Ref | Ref | Ref | Ref | Ref | Ref | Ref | Ref | Ref | Ref | Ref | Ref | Ref | Ref | Ref | Ref | Ref | Ref | Ref | Ref |
| Below Poverty threshold | 1.78 | 1.33, 2.36 | **<0.001** | 1.79 | 1.34, 2.39 | **<0.001** | 1.79 | 1.35, 2.39 | **<0.001** | 1.79 | 1.34, 2.39 | **<0.001** | 1.79 | 1.34, 2.40 | **<0.001** | 1.8 | 1.35, 2.40 | **<0.001** | 1.87 | 1.31, 2.66 | **<0.001** | 1.84 | 1.29, 2.62 | **<0.001** | 1.83 | 1.28, 2.62 | **<0.001** |
| **APOE E4 status** |  |  |  |  |  |  |  |  |  |  |  |  |  |  |  |  |  |  |  |  |  |  |  |  |  |  |  |
| No copies of e4 | Ref | Ref | Ref | Ref | Ref | Ref | Ref | Ref | Ref | Ref | Ref | Ref | Ref | Ref | Ref | Ref | Ref | Ref | Ref | Ref | Ref | Ref | Ref | Ref | Ref | Ref | Ref |
| Any copies of e4 | 1.02 | 0.80, 1.29 | 0.900 | 1.02 | 0.80, 1.29 | >0.9 | 1.01 | 0.80, 1.28 | >0.9 | 1 | 0.79, 1.27 | >0.9 | 1.00 | 0.79, 1.27 | >0.9 | 1 | 0.79, 1.27 | >0.9 | 1.44 | 1.05, 1.96 | **0.024** | 1.45 | 1.06, 1.99 | **0.020** | 1.45 | 1.06, 1.99 | **0.021** |
| **Social Ladder** | 0.97 | 0.91, 1.03 | 0.300 | 0.96 | 0.89, 1.02 | 0.200 | 0.96 | 0.90, 1.02 | **0.2** | 0.97 | 0.91, 1.04 | 0.400 | 0.96 | 0.90, 1.03 | 0.200 | 0.96 | 0.90, 1.03 | **0.3** | 1.07 | 0.98, 1.16 | 0.150 | 1.06 | 0.98, 1.16 | 0.200 | 1.06 | 0.98, 1.16 | **0.2** |
| **Baseline wave** |  |  |  |  |  |  |  |  |  |  |  |  |  |  |  |  |  |  |  |  |  |  |  |  |  |  |  |
| Wave 1 (2008) | Ref | Ref | Ref | Ref | Ref | Ref | Ref | Ref | Ref | Ref | Ref | Ref | Ref | Ref | Ref | Ref | Ref | Ref | Ref | Ref | Ref | Ref | Ref | Ref | Ref | Ref | Ref |
| Wave 2 (2010) | 1.19 | 0.93, 1.51 | 0.200 | 1.17 | 0.92, 1.49 | 0.200 | 1.16 | 0.91, 1.48 | 0.2 | 1.19 | 0.93, 1.52 | 0.200 | 1.18 | 0.92, 1.50 | 0.200 | 1.17 | 0.91, 1.49 | 0.2 | 1.12 | 0.82, 1.52 | 0.500 | 1.11 | 0.81, 1.52 | 0.500 | 1.11 | 0.81, 1.52 | 0.5 |
| **PGS-AD** |  |  |  |  |  |  |  |  |  |  |  |  |  |  |  |  |  |  |  |  |  |  |  |  |  |  |  |
| Below 75% | - | - | - | Ref | Ref | Ref | Ref | Ref | Ref | - | - | - | Ref | Ref | Ref | Ref | Ref | Ref | - | - | - | Ref | Ref | Ref | Ref | Ref | Ref |
| Above 75% | - | - | - | 1.11 | 0.84, 1.48 | 0.500 | 1.39 | 0.91, 2.13 | 0.13 | - | - | - | 1.07 | 0.80, 1.43 | 0.700 | 1.28 | 0.83, 1.98 | 0.3 | - | - | - | 0.98 | 0.65, 1.47 | >0.9 | 1.05 | 0.58, 1.90 | 0.9 |
| **Neighborhood* PGS-AD** | - | - | - | - | - | - | 0.7 | 0.42, 1.17 | 0.2 | - | - | - | - | - | - | 0.75 | 0.44, 1.27 | 0.3 | - | - | - | - | - | - | 0.88 | 0.42, 1.86 | 0.7 |
| RERI: The most disadvantaged neighborhoods*PGS-AD Above 75% | - | - | - | - | - | - | -0.42 | -1.09, 0.25 |  | - | - | - | - | - | - | -0.33 | -0.97, 0.31 |  | - | - | - | - | - | - | -0.14 | -0.96, 0.68 |  |
