# Supplementary material for: Associations of perceived neighborhood factors and Alzheimer’s disease polygenic score with cognition: Evidence from the Health and Retirement Study
Source: PLoS One. 2025 Nov 20;20(11):e0336403. doi: 10.1371/journal.pone.0336403 (PMC12633890; doi:10.1371/journal.pone.0336403)
Supplement: S4 Table — Each model further adjusted for Smoking, Alcohol Consumption, BMI, Diabetes, Self-Rated Hearing and Vision, Brain-related Conditions, Chronic Conditions, and Depression. (DOCX) [file pone.0336403.s004.docx]

**Supplemental Table 4.** Hazard Ratios from survival analysis with additional sensitivity covariates stratified by Ancestry, estimates present the association for each standard deviation increase in the neighborhood disadvantage index with incident cognitive impairment (CIND and dementia), CIND and dementia, relative to normal cognition and non-dementia respectively in the US Health and Retirement Study (2008-2010 Waves).

|  | **Cognitive Impairment vs. Normal Cognition, European Ancestry (n=6,031)** | | | | | | | | | **CIND vs. Normal Cognition, European Ancestry (n=5,962)** | | | | | | | | | **Dementia vs. Non-dementia, European Ancestry (n=6,685)** | | | | | | | | |
| --- | --- | --- | --- | --- | --- | --- | --- | --- | --- | --- | --- | --- | --- | --- | --- | --- | --- | --- | --- | --- | --- | --- | --- | --- | --- | --- | --- |
|  | **Model 1** | | | **Model 2** | | | **Model 3** | | | **Model 1** | | | **Model 2** | | | **Model 3** | | | **Model 1** | | | **Model 2** | | | **Model 3** | | |
|  | **HR** | **95% CI** | **p-value** | **HR** | **95% CI** | **p-value** | **HR** | **95% CI** | **p-value** | **HR** | **95% CI** | **p-value** | **HR** | **95% CI** | **p-value** | **HR** | **95% CI** | **p-value** | **HR** | **95% CI** | **p-value** | **HR** | **95% CI** | **p-value** | **HR** | **95% CI** | **p-value** |
| **Neighborhood** | 1.06 | 1.00, 1.11 | **0.048** | 1.06 | 1.00, 1.12 | **0.044** | 1.06 | 1.00, 1.12 | **0.045** | 1.05 | 1.00, 1.11 | 0.069 | 1.05 | 1.00, 1.11 | 0.068 | 1.05 | 1.00, 1.11 | 0.068 | 1.11 | 1.01, 1.22 | **0.036** | 1.11 | 1.00, 1.22 | **0.041** | 1.11 | 1.00, 1.22 | **0.043** |
| **Age** | 1.08 | 1.07, 1.08 | **<0.001** | 1.08 | 1.07, 1.08 | **<0.001** | 1.08 | 1.07, 1.08 | **<0.001** | 1.08 | 1.07, 1.08 | **<0.001** | 1.08 | 1.07, 1.08 | **<0.001** | 1.08 | 1.07, 1.08 | **<0.001** | 1.11 | 1.10, 1.13 | **<0.001** | 1.11 | 1.10, 1.13 | **<0.001** | 1.11 | 1.10, 1.13 | **<0.001** |
| **Sex** |  |  |  |  |  |  |  |  |  |  |  |  |  |  |  |  |  |  |  |  |  |  |  |  |  |  |  |
| Female | Ref | Ref | Ref | Ref | Ref | Ref | Ref | Ref | Ref | Ref | Ref | Ref | Ref | Ref | Ref | Ref | Ref | Ref | Ref | Ref | Ref | Ref | Ref | Ref | Ref | Ref | Ref |
| Male | 1.25 | 1.13, 1.39 | **<0.001** | 1.25 | 1.12, 1.38 | **<0.001** | 1.25 | 1.12, 1.38 | **<0.001** | 1.26 | 1.13, 1.40 | **<0.001** | 1.25 | 1.13, 1.39 | **<0.001** | 1.25 | 1.13, 1.39 | **<0.001** | 1.14 | 0.93, 1.41 | 0.200 | 1.15 | 0.93, 1.42 | 0.200 | 1.15 | 0.93, 1.42 | 0.2 |
| **Education** |  |  |  |  |  |  |  |  |  |  |  |  |  |  |  |  |  |  |  |  |  |  |  |  |  |  |  |
| Above High School/GED | Ref | Ref | Ref | Ref | Ref | Ref | Ref | Ref | Ref | Ref | Ref | Ref | Ref | Ref | Ref | Ref | Ref | Ref | Ref | Ref | Ref | Ref | Ref | Ref | Ref | Ref | Ref |
| High School/GED | 1.58 | 1.41, 1.78 | **<0.001** | 1.59 | 1.41, 1.79 | **<0.001** | 1.59 | 1.41, 1.79 | **<0.001** | 1.61 | 1.43, 1.82 | **<0.001** | 1.61 | 1.43, 1.82 | **<0.001** | 1.61 | 1.43, 1.82 | **<0.001** | 1.65 | 1.27, 2.14 | **<0.001** | 1.66 | 1.28, 2.15 | **<0.001** | 1.66 | 1.28, 2.15 | **<0.001** |
| Less than High School/GED | 2.78 | 2.34, 3.30 | **<0.001** | 2.79 | 2.35, 3.31 | **<0.001** | 2.78 | 2.35, 3.30 | **<0.001** | 2.83 | 2.38, 3.38 | **<0.001** | 2.84 | 2.39, 3.39 | **<0.001** | 2.84 | 2.39, 3.38 | **<0.001** | 3.96 | 2.91, 5.40 | **<0.001** | 4.01 | 2.95, 5.47 | **<0.001** | 4.01 | 2.94, 5.46 | **<0.001** |
| **Poverty Status** |  |  |  |  |  |  |  |  |  |  |  |  |  |  |  |  |  |  |  |  |  |  |  |  |  |  |  |
| Above Poverty threshold | Ref | Ref | Ref | Ref | Ref | Ref | Ref | Ref | Ref | Ref | Ref | Ref | Ref | Ref | Ref | Ref | Ref | Ref | Ref | Ref | Ref | Ref | Ref | Ref | Ref | Ref | Ref |
| Below Poverty threshold | 1.15 | 0.89, 1.49 | 0.300 | 1.15 | 0.88, 1.49 | 0.300 | 1.15 | 0.88, 1.49 | 0.300 | 1.16 | 0.90, 1.51 | 0.300 | 1.16 | 0.89, 1.51 | 0.300 | 1.16 | 0.89, 1.51 | 0.300 | 1.79 | 1.20, 2.67 | **0.004** | 1.84 | 1.23, 2.74 | **0.003** | 1.84 | 1.23, 2.74 | **0.003** |
| **APOE E4 status** |  |  |  |  |  |  |  |  |  |  |  |  |  |  |  |  |  |  |  |  |  |  |  |  |  |  |  |
| No copies of e4 | Ref | Ref | Ref | Ref | Ref | Ref | Ref | Ref | Ref | Ref | Ref | Ref | Ref | Ref | Ref | Ref | Ref | Ref | Ref | Ref | Ref | Ref | Ref | Ref | Ref | Ref | Ref |
| Any copies of e4 | 1.44 | 1.30, 1.60 | **<0.001** | 1.42 | 1.28, 1.58 | **<0.001** | 1.42 | 1.28, 1.58 | **<0.001** | 1.45 | 1.30, 1.61 | **<0.001** | 1.43 | 1.29, 1.60 | **<0.001** | 1.43 | 1.29, 1.60 | **<0.001** | 2.06 | 1.70, 2.51 | **<0.001** | 2.05 | 1.69, 2.50 | **<0.001** | 2.05 | 1.68, 2.50 | **<0.001** |
| **Social Ladder** | 0.96 | 0.93, 0.99 | **0.008** | 0.96 | 0.93, 0.99 | **0.009** | 0.96 | 0.93, 0.99 | **0.009** | 0.96 | 0.93, 0.99 | **0.006** | 0.96 | 0.93, 0.99 | **0.006** | 0.96 | 0.93, 0.99 | **0.006** | 1.01 | 0.95, 1.08 | 0.700 | 1.01 | 0.95, 1.08 | 0.700 | 1.01 | 0.95, 1.08 | 0.700 |
| **Baseline wave** |  |  |  |  |  |  |  |  |  |  |  |  |  |  |  |  |  |  |  |  |  |  |  |  |  |  |  |
| Wave 1 (2008) | Ref | Ref | Ref | Ref | Ref | Ref | Ref | Ref | Ref | Ref | Ref | Ref | Ref | Ref | Ref | Ref | Ref | Ref | Ref | Ref | Ref | Ref | Ref | Ref | Ref | Ref | Ref |
| Wave 2 (2010) | 0.86 | 0.78, 0.95 | **0.002** | 0.87 | 0.79, 0.95 | **0.003** | 0.87 | 0.79, 0.95 | **0.003** | 0.86 | 0.79, 0.95 | **0.003** | 0.87 | 0.79, 0.96 | **0.005** | 0.87 | 0.79, 0.96 | **0.005** | 0.81 | 0.67, 0.98 | **0.027** | 0.81 | 0.67, 0.98 | **0.028** | 0.81 | 0.67, 0.98 | **0.027** |
| **PGS-AD** | - | - | - | 1.10 | 1.05, 1.16 | **<0.001** | 1.10 | 1.05, 1.16 | **<0.001** | - | - | - | 1.10 | 1.05, 1.16 | **<0.001** | 1.10 | 1.04, 1.16 | **<0.001** | - | - | - | 1.05 | 0.95, 1.15 | 0.400 | 1.04 | 0.94, 1.15 | 0.400 |
| **Neighborhood* PGS-AD** | - | - | - | - | - | - | 0.99 | 0.94, 1.04 | 0.7 | - | - | - | - | - | - | 0.99 | 0.94, 1.05 | 0.800 | - | - | - | - | - | - | 0.98 | 0.89, 1.08 | 0.700 |
| **Smoking status** |  |  |  |  |  |  |  |  |  |  |  |  |  |  |  |  |  |  |  |  |  |  |  |  |  |  |  |
| Never Smoker | Ref | Ref | Ref | Ref | Ref | Ref | Ref | Ref | Ref | Ref | Ref | Ref | Ref | Ref | Ref | Ref | Ref | Ref | Ref | Ref | Ref | Ref | Ref | Ref | Ref | Ref | Ref |
| Current Smoker | 1.23 | 1.04, 1.47 | **0.017** | 1.24 | 1.04, 1.47 | **0.015** | 1.24 | 1.04, 1.47 | **0.015** | 1.24 | 1.04, 1.48 | **0.016** | 1.25 | 1.05, 1.48 | **0.014** | 1.25 | 1.05, 1.48 | **0.014** | 1.01 | 0.69, 1.48 | >0.9 | 1.00 | 0.68, 1.47 | >0.9 | 1.00 | 0.68, 1.47 | >0.9 |
| Former Smoke | 1.06 | 0.96, 1.18 | 0.200 | 1.06 | 0.95, 1.17 | 0.300 | 1.06 | 0.95, 1.17 | 0.300 | 1.06 | 0.96, 1.18 | 0.300 | 1.05 | 0.95, 1.17 | 0.400 | 1.05 | 0.95, 1.17 | 0.400 | 0.98 | 0.80, 1.20 | 0.900 | 0.98 | 0.80, 1.20 | 0.800 | 0.98 | 0.80, 1.20 | 0.800 |
| **Drinking (# drinks/day when drinks)** | 1.01 | 0.97, 1.05 | 0.800 | 1.01 | 0.97, 1.05 | 0.700 | 1.01 | 0.97, 1.05 | 0.700 | 1.01 | 0.97, 1.05 | 0.700 | 1.01 | 0.97, 1.05 | 0.600 | 1.01 | 0.97, 1.05 | 0.600 | 1.02 | 0.93, 1.11 | 0.700 | 1.02 | 0.93, 1.12 | 0.700 | 1.02 | 0.93, 1.12 | 0.700 |
| **Depression** | 1.08 | 1.05, 1.11 | **<0.001** | 1.08 | 1.05, 1.11 | **<0.001** | 1.08 | 1.05, 1.11 | **<0.001** | 1.08 | 1.05, 1.11 | **<0.001** | 1.08 | 1.05, 1.11 | **<0.001** | 1.08 | 1.05, 1.11 | **<0.001** | 1.08 | 1.02, 1.14 | **0.004** | 1.08 | 1.03, 1.14 | **0.004** | 1.08 | 1.03, 1.14 | **0.004** |
| **BMI** | 1.00 | 0.99, 1.00 | 0.300 | 1.00 | 0.99, 1.01 | 0.300 | 1.00 | 0.99, 1.01 | 0.4 | 1.00 | 0.99, 1.01 | 0.4 | 1.00 | 0.99, 1.01 | 0.600 | 1.00 | 0.99, 1.01 | 0.600 | 0.97 | 0.95, 0.99 | **0.006** | 0.97 | 0.95, 0.99 | **0.006** | 0.97 | 0.95, 0.99 | **0.006** |
| **Ever have Diabetes** |  |  |  |  |  |  |  |  |  |  |  |  |  |  |  |  |  |  |  |  |  |  |  |  |  |  |  |
| No | Ref | Ref | Ref | Ref | Ref | Ref | Ref | Ref | Ref | Ref | Ref | Ref | Ref | Ref | Ref | Ref | Ref | Ref | Ref | Ref | Ref | Ref | Ref | Ref | Ref | Ref | Ref |
| Yes | 1.17 | 1.01, 1.34 | **0.030** | 1.15 | 1.00, 1.32 | **0.049** | 1.15 | 1.00, 1.32 | **0.049** | 1.19 | 1.03, 1.37 | **0.017** | 1.17 | 1.02, 1.35 | **0.028** | 1.17 | 1.02, 1.35 | **0.028** | 1.15 | 0.87, 1.53 | 0.3 | 1.15 | 0.86, 1.52 | 0.300 | 1.15 | 0.87, 1.53 | 0.300 |
| **Brain Condition** |  |  |  |  |  |  |  |  |  |  |  |  |  |  |  |  |  |  |  |  |  |  |  |  |  |  |  |
| No | Ref | Ref | Ref | Ref | Ref | Ref | Ref | Ref | Ref | Ref | Ref | Ref | Ref | Ref | Ref | Ref | Ref | Ref | Ref | Ref | Ref | Ref | Ref | Ref | Ref | Ref | Ref |
| Yes | 1.16 | 1.03, 1.31 | **0.014** | 1.15 | 1.02, 1.30 | **0.022** | 1.15 | 1.02, 1.30 | **0.022** | 1.15 | 1.02, 1.30 | **0.027** | 1.14 | 1.01, 1.29 | **0.040** | 1.14 | 1.01, 1.29 | **0.040** | 1.49 | 1.19, 1.87 | **<0.001** | 1.49 | 1.19, 1.87 | **<0.001** | 1.49 | 1.19, 1.87 | **<0.001** |
| **Chronic Condition** |  |  |  |  |  |  |  |  |  |  |  |  |  |  |  |  |  |  |  |  |  |  |  |  |  |  |  |
| None | Ref | Ref | Ref | Ref | Ref | Ref | Ref | Ref | Ref | Ref | Ref | Ref | Ref | Ref | Ref | Ref | Ref | Ref | Ref | Ref | Ref | Ref | Ref | Ref | Ref | Ref | Ref |
| 1 - 2 | 0.95 | 0.81, 1.12 | 0.600 | 0.95 | 0.81, 1.12 | 0.600 | 0.95 | 0.82, 1.12 | 0.600 | 0.97 | 0.83, 1.14 | 0.700 | 0.97 | 0.83, 1.14 | 0.700 | 0.97 | 0.83, 1.14 | 0.700 | 0.74 | 0.55, 1.01 | 0.057 | 0.74 | 0.54, 1.01 | 0.055 | 0.74 | 0.54, 1.01 | 0.055 |
| >= 3 | 1.02 | 0.85, 1.23 | 0.800 | 1.04 | 0.86, 1.25 | 0.700 | 1.04 | 0.86, 1.25 | 0.700 | 1.03 | 0.86, 1.25 | 0.700 | 1.05 | 0.87, 1.27 | 0.600 | 1.05 | 0.87, 1.27 | 0.600 | 0.63 | 0.44, 0.90 | **0.011** | 0.62 | 0.44, 0.89 | **0.010** | 0.62 | 0.44, 0.89 | **0.010** |
| **Eyesight** |  |  |  |  |  |  |  |  |  |  |  |  |  |  |  |  |  |  |  |  |  |  |  |  |  |  |  |
| Excellent | Ref | Ref | Ref | Ref | Ref | Ref | Ref | Ref | Ref | Ref | Ref | Ref | Ref | Ref | Ref | Ref | Ref | Ref | Ref | Ref | Ref | Ref | Ref | Ref | Ref | Ref | Ref |
| Very Good | 0.94 | 0.78, 1.13 | 0.5 | 0.94 | 0.78, 1.13 | 0.500 | 0.94 | 0.78, 1.13 | 0.500 | 0.95 | 0.79, 1.15 | 0.600 | 0.95 | 0.79, 1.15 | 0.600 | 0.95 | 0.79, 1.15 | 0.600 | 0.66 | 0.46, 0.96 | **0.031** | 0.67 | 0.46, 0.97 | **0.032** | 0.66 | 0.46, 0.96 | **0.031** |
| Good | 1.02 | 0.85, 1.22 | 0.9 | 1.02 | 0.85, 1.22 | 0.900 | 1.02 | 0.85, 1.22 | 0.900 | 1.02 | 0.85, 1.23 | 0.800 | 1.02 | 0.85, 1.23 | 0.800 | 1.02 | 0.85, 1.23 | 0.800 | 0.87 | 0.61, 1.24 | 0.400 | 0.87 | 0.61, 1.23 | 0.400 | 0.87 | 0.61, 1.23 | 0.400 |
| Fair | 1.30 | 1.06, 1.60 | **0.013** | 1.29 | 1.05, 1.59 | **0.016** | 1.29 | 1.05, 1.59 | **0.016** | 1.31 | 1.06, 1.62 | **0.013** | 1.30 | 1.05, 1.61 | **0.016** | 1.30 | 1.05, 1.61 | **0.015** | 0.93 | 0.62, 1.38 | 0.700 | 0.91 | 0.61, 1.36 | 0.600 | 0.91 | 0.61, 1.36 | 0.600 |
| Poor | 1.06 | 0.78, 1.43 | 0.7 | 1.03 | 0.76, 1.40 | 0.800 | 1.04 | 0.76, 1.40 | 0.800 | 1.06 | 0.78, 1.45 | 0.700 | 1.04 | 0.76, 1.42 | 0.800 | 1.04 | 0.76, 1.42 | 0.800 | 1.23 | 0.76, 2.00 | 0.400 | 1.20 | 0.74, 1.96 | 0.500 | 1.20 | 0.74, 1.95 | 0.500 |
| Blind | 0.78 | 0.25, 2.46 | 0.7 | 0.72 | 0.23, 2.27 | 0.600 | 0.72 | 0.23, 2.27 | 0.600 | 0.60 | 0.15, 2.43 | 0.5 | 0.55 | 0.13, 2.22 | 0.400 | 0.54 | 0.13, 2.22 | 0.400 | 1.49 | 0.35, 6.26 | 0.600 | 1.45 | 0.34, 6.12 | 0.600 | 1.44 | 0.34, 6.10 | 0.600 |
| **Hearing** |  |  |  |  |  |  |  |  |  |  |  |  |  |  |  |  |  |  |  |  |  |  |  |  |  |  |  |
| Excellent | Ref | Ref | Ref | Ref | Ref | Ref | Ref | Ref | Ref | Ref | Ref | Ref | Ref | Ref | Ref | Ref | Ref | Ref | Ref | Ref | Ref | Ref | Ref | Ref | Ref | Ref | Ref |
| Very Good | 0.94 | 0.80, 1.10 | 0.400 | 0.93 | 0.80, 1.09 | 0.400 | 0.93 | 0.80, 1.09 | 0.400 | 0.93 | 0.79, 1.09 | 0.400 | 0.93 | 0.79, 1.09 | 0.400 | 0.93 | 0.79, 1.09 | 0.300 | 1.09 | 0.78, 1.51 | 0.600 | 1.10 | 0.79, 1.53 | 0.600 | 1.10 | 0.78, 1.53 | 0.600 |
| Good | 1.04 | 0.89, 1.22 | 0.600 | 1.04 | 0.89, 1.21 | 0.600 | 1.04 | 0.89, 1.21 | 0.600 | 1.04 | 0.89, 1.22 | 0.600 | 1.03 | 0.88, 1.21 | 0.700 | 1.03 | 0.88, 1.21 | 0.700 | 1.08 | 0.78, 1.48 | 0.600 | 1.08 | 0.79, 1.48 | 0.600 | 1.08 | 0.78, 1.48 | 0.600 |
| Fair | 1.24 | 1.04, 1.48 | **0.016** | 1.22 | 1.03, 1.46 | **0.025** | 1.22 | 1.02, 1.46 | **0.026** | 1.24 | 1.04, 1.48 | **0.019** | 1.22 | 1.02, 1.46 | **0.028** | 1.22 | 1.02, 1.46 | **0.029** | 1.29 | 0.91, 1.84 | 0.150 | 1.31 | 0.92, 1.86 | 0.130 | 1.31 | 0.92, 1.86 | 0.130 |
| Poor | 1.32 | 1.04, 1.68 | **0.020** | 1.30 | 1.03, 1.65 | **0.030** | 1.30 | 1.02, 1.65 | **0.031** | 1.34 | 1.05, 1.71 | **0.017** | 1.31 | 1.03, 1.67 | **0.026** | 1.31 | 1.03, 1.67 | **0.027** | 1.13 | 0.72, 1.76 | 0.600 | 1.13 | 0.72, 1.77 | 0.600 | 1.13 | 0.72, 1.77 | 0.600 |
|  | **Cognitive Impairment vs. Normal Cognition, African Ancestry (n=696)** | | | | | | | | | **CIND vs. Normal Cognition, African Ancestry (n=688)** | | | | | | | | | **Dementia vs. Non-dementia, African Ancestry (n=960)** | | | | | | | | |
|  | **Model 1** | | | **Model 2** | | | **Model 3** | | | **Model 1** | | | **Model 2** | | | **Model 3** | | | **Model 1** | | | **Model 2** | | | **Model 3** | | |
|  | **HR** | **95% CI** | **p-value** | **HR** | **95% CI** | **p-value** | **HR** | **95% CI** | **p-value** | **HR** | **95% CI** | **p-value** | **HR** | **95% CI** | **p-value** | **HR** | **95% CI** | **p-value** | **HR** | **95% CI** | **p-value** | **HR** | **95% CI** | **p-value** | **HR** | **95% CI** | **p-value** |
| **Neighborhood** | 1.00 | 0.90, 1.12 | >0.9 | 1.00 | 0.89, 1.12 | >0.9 | 1.01 | 0.90, 1.13 | >0.9 | 1.00 | 0.89, 1.11 | >0.9 | 0.99 | 0.89, 1.11 | >0.9 | 1.00 | 0.89, 1.12 | >0.9 | 1.02 | 0.88, 1.18 | 0.800 | 1.02 | 0.89, 1.19 | 0.700 | 1.03 | 0.89, 1.19 | 0.700 |
| **Age** | 1.06 | 1.04, 1.07 | **<0.001** | 1.06 | 1.04, 1.08 | **<0.001** | 1.06 | 1.04, 1.08 | **<0.001** | 1.06 | 1.04, 1.08 | **<0.001** | 1.06 | 1.05, 1.08 | **<0.001** | 1.06 | 1.05, 1.08 | **<0.001** | 1.10 | 1.07, 1.12 | **<0.001** | 1.10 | 1.08, 1.12 | **<0.001** | 1.10 | 1.08, 1.12 | **<0.001** |
| **Sex** |  |  |  |  |  |  |  |  |  |  |  |  |  |  |  |  |  |  |  |  |  |  |  |  |  |  |  |
| Female | Ref | Ref | Ref | Ref | Ref | Ref | Ref | Ref | Ref | Ref | Ref | Ref | Ref | Ref | Ref | Ref | Ref | Ref | Ref | Ref | Ref | Ref | Ref | Ref | Ref | Ref | Ref |
| Male | 1.15 | 0.89, 1.49 | 0.300 | 1.15 | 0.89, 1.50 | 0.300 | 1.16 | 0.89, 1.51 | 0.300 | 1.16 | 0.89, 1.50 | 0.300 | 1.16 | 0.89, 1.50 | 0.300 | 1.16 | 0.89, 1.51 | 0.300 | 1.31 | 0.92, 1.85 | 0.130 | 1.36 | 0.96, 1.94 | 0.085 | 1.36 | 0.96, 1.94 | 0.084 |
| **Education** |  |  |  |  |  |  |  |  |  |  |  |  |  |  |  |  |  |  |  |  |  |  |  |  |  |  |  |
| Above High School/GED | Ref | Ref | Ref | Ref | Ref | Ref | Ref | Ref | Ref | Ref | Ref | Ref | Ref | Ref | Ref | Ref | Ref | Ref | Ref | Ref | Ref | Ref | Ref | Ref | Ref | Ref | Ref |
| High School/GED | 1.47 | 1.08, 2.01 | **0.014** | 1.47 | 1.07, 2.00 | **0.016** | 1.46 | 1.07, 1.99 | **0.017** | 1.50 | 1.09, 2.06 | **0.012** | 1.49 | 1.08, 2.05 | **0.015** | 1.48 | 1.08, 2.04 | **0.016** | 2.16 | 1.16, 4.03 | **0.015** | 2.30 | 1.23, 4.30 | **0.009** | 2.30 | 1.23, 4.30 | **0.009** |
| Less than High School/GED | 3.00 | 2.09, 4.31 | **<0.001** | 2.87 | 2.00, 4.13 | **<0.001** | 2.90 | 2.01, 4.17 | **<0.001** | 3.11 | 2.15, 4.50 | **<0.001** | 2.95 | 2.04, 4.27 | **<0.001** | 2.97 | 2.05, 4.30 | **<0.001** | 5.21 | 2.77, 9.80 | **<0.001** | 5.59 | 2.95, 10.6 | **<0.001** | 5.60 | 2.96, 10.6 | **<0.001** |
| **Poverty Status** |  |  |  |  |  |  |  |  |  |  |  |  |  |  |  |  |  |  |  |  |  |  |  |  |  |  |  |
| Above Poverty threshold | Ref | Ref | Ref | Ref | Ref | Ref | Ref | Ref | Ref | Ref | Ref | Ref | Ref | Ref | Ref | Ref | Ref | Ref | Ref | Ref | Ref | Ref | Ref | Ref | Ref | Ref | Ref |
| Below Poverty threshold | 1.46 | 1.08, 1.99 | **0.014** | 1.48 | 1.09, 2.01 | **0.012** | 1.49 | 1.10, 2.03 | **0.011** | 1.46 | 1.08, 1.99 | **0.015** | 1.47 | 1.08, 2.00 | **0.015** | 1.48 | 1.09, 2.02 | **0.013** | 1.53 | 1.04, 2.24 | **0.029** | 1.49 | 1.02, 2.19 | **0.040** | 1.50 | 1.02, 2.20 | **0.039** |
| **APOE E4 status** |  |  |  |  |  |  |  |  |  |  |  |  |  |  |  |  |  |  |  |  |  |  |  |  |  |  |  |
| No copies of e4 | Ref | Ref | Ref | Ref | Ref | Ref | Ref | Ref | Ref | Ref | Ref | Ref | Ref | Ref | Ref | Ref | Ref | Ref | Ref | Ref | Ref | Ref | Ref | Ref | Ref | Ref | Ref |
| Any copies of e4 | 0.91 | 0.71, 1.16 | 0.400 | 0.91 | 0.71, 1.17 | 0.500 | 0.90 | 0.70, 1.15 | 0.400 | 0.89 | 0.69, 1.14 | 0.400 | 0.89 | 0.69, 1.15 | 0.400 | 0.88 | 0.68, 1.14 | 0.300 | 1.39 | 1.01, 1.93 | **0.044** | 1.43 | 1.03, 1.98 | **0.033** | 1.40 | 1.00, 1.94 | **0.047** |
| **Social Ladder** | 0.99 | 0.93, 1.05 | 0.700 | 0.98 | 0.91, 1.04 | 0.500 | 0.98 | 0.91, 1.04 | 0.500 | 0.99 | 0.93, 1.06 | 0.800 | 0.98 | 0.92, 1.05 | 0.600 | 0.98 | 0.92, 1.05 | 0.600 | 1.09 | 1.00, 1.19 | 0.055 | 1.09 | 1.00, 1.19 | 0.057 | 1.09 | 1.00, 1.19 | 0.055 |
| **Baseline wave** |  |  |  |  |  |  |  |  |  |  |  |  |  |  |  |  |  |  |  |  |  |  |  |  |  |  |  |
| Wave 1 (2008) | Ref | Ref | Ref | Ref | Ref | Ref | Ref | Ref | Ref | Ref | Ref | Ref | Ref | Ref | Ref | Ref | Ref | Ref | Ref | Ref | Ref | Ref | Ref | Ref | Ref | Ref | Ref |
| Wave 2 (2010) | 1.15 | 0.90, 1.47 | 0.300 | 1.13 | 0.88, 1.44 | 0.300 | 1.12 | 0.88, 1.44 | 0.400 | 1.16 | 0.91, 1.48 | 0.200 | 1.13 | 0.88, 1.46 | 0.300 | 1.13 | 0.88, 1.45 | 0.300 | 1.09 | 0.79, 1.50 | 0.600 | 1.05 | 0.76, 1.46 | 0.800 | 1.05 | 0.76, 1.46 | 0.800 |
| **PGS-AD** | - | - | - | 1.13 | 0.94, 1.35 | 0.200 | 1.15 | 0.96, 1.37 | 0.140 | - | - | - | 1.12 | 0.94, 1.34 | 0.200 | 1.13 | 0.94, 1.36 | 0.200 | - | - | - | 1.10 | 0.86, 1.41 | 0.400 | 1.13 | 0.88, 1.46 | 0.300 |
| **Neighborhood* PGS-AD** | - | - | - | - | - | - | 0.95 | 0.83, 1.08 | 0.4 | - | - | - | - | - | - | 0.95 | 0.84, 1.09 | 0.500 | - | - | - | - | - | - | 0.92 | 0.78, 1.09 | 0.300 |
| **Smoking status** |  |  |  |  |  |  |  |  |  |  |  |  |  |  |  |  |  |  |  |  |  |  |  |  |  |  |  |
| Never Smoker | Ref | Ref | Ref | Ref | Ref | Ref | Ref | Ref | Ref | Ref | Ref | Ref | Ref | Ref | Ref | Ref | Ref | Ref | Ref | Ref | Ref | Ref | Ref | Ref | Ref | Ref | Ref |
| Current Smoker | 1.24 | 0.88, 1.74 | 0.200 | 1.25 | 0.89, 1.76 | 0.200 | 1.25 | 0.89, 1.75 | 0.200 | 1.27 | 0.90, 1.79 | 0.200 | 1.28 | 0.91, 1.81 | 0.200 | 1.28 | 0.91, 1.80 | 0.200 | 1.34 | 0.79, 2.27 | 0.300 | 1.36 | 0.80, 2.31 | 0.300 | 1.37 | 0.80, 2.33 | 0.200 |
| Former Smoke | 0.98 | 0.75, 1.28 | >0.9 | 1.03 | 0.79, 1.35 | 0.800 | 1.03 | 0.78, 1.35 | 0.800 | 0.96 | 0.73, 1.25 | 0.700 | 0.99 | 0.75, 1.30 | >0.9 | 0.99 | 0.75, 1.30 | >0.9 | 1.43 | 1.00, 2.04 | **0.048** | 1.44 | 1.01, 2.07 | **0.045** | 1.44 | 1.00, 2.06 | **0.048** |
| **Drinking (# drinks/day when drinks)** | 1.04 | 0.95, 1.14 | 0.400 | 1.04 | 0.95, 1.14 | 0.400 | 1.04 | 0.95, 1.14 | 0.400 | 1.04 | 0.95, 1.15 | 0.400 | 1.04 | 0.95, 1.15 | 0.400 | 1.04 | 0.95, 1.15 | 0.400 | 1.00 | 0.87, 1.15 | >0.9 | 1.00 | 0.87, 1.14 | >0.9 | 1.00 | 0.87, 1.14 | >0.9 |
| **Depression** | 1.07 | 1.00, 1.13 | **0.041** | 1.07 | 1.00, 1.13 | **0.048** | 1.06 | 1.00, 1.13 | **0.050** | 1.07 | 1.01, 1.14 | **0.031** | 1.07 | 1.00, 1.14 | **0.035** | 1.07 | 1.00, 1.14 | **0.037** | 1.12 | 1.03, 1.21 | **0.005** | 1.12 | 1.04, 1.22 | **0.003** | 1.13 | 1.04, 1.22 | **0.003** |
| **BMI** | 0.99 | 0.97, 1.01 | 0.400 | 0.99 | 0.97, 1.01 | 0.400 | 0.99 | 0.97, 1.01 | 0.400 | 0.99 | 0.98, 1.01 | 0.500 | 0.99 | 0.98, 1.01 | 0.500 | 0.99 | 0.98, 1.01 | 0.500 | 0.99 | 0.97, 1.02 | 0.500 | 0.99 | 0.97, 1.02 | 0.500 | 0.99 | 0.97, 1.02 | 0.500 |
| **Ever have Diabetes** |  |  |  |  |  |  |  |  |  |  |  |  |  |  |  |  |  |  |  |  |  |  |  |  |  |  |  |
| No | Ref | Ref | Ref | Ref | Ref | Ref | Ref | Ref | Ref | Ref | Ref | Ref | Ref | Ref | Ref | Ref | Ref | Ref | Ref | Ref | Ref | Ref | Ref | Ref | Ref | Ref | Ref |
| Yes | 1.14 | 0.83, 1.55 | 0.400 | 1.17 | 0.86, 1.61 | 0.300 | 1.17 | 0.85, 1.60 | 0.300 | 1.15 | 0.84, 1.58 | 0.400 | 1.18 | 0.86, 1.63 | 0.300 | 1.18 | 0.86, 1.62 | 0.300 | 0.93 | 0.61, 1.42 | 0.700 | 0.98 | 0.64, 1.51 | >0.9 | 0.97 | 0.63, 1.49 | >0.9 |
| **Brain Condition** |  |  |  |  |  |  |  |  |  |  |  |  |  |  |  |  |  |  |  |  |  |  |  |  |  |  |  |
| No | Ref | Ref | Ref | Ref | Ref | Ref | Ref | Ref | Ref | Ref | Ref | Ref | Ref | Ref | Ref | Ref | Ref | Ref | Ref | Ref | Ref | Ref | Ref | Ref | Ref | Ref | Ref |
| Yes | 1.32 | 0.98, 1.79 | 0.071 | 1.36 | 1.00, 1.85 | **0.049** | 1.36 | 1.00, 1.85 | **0.049** | 1.32 | 0.97, 1.80 | 0.078 | 1.36 | 0.99, 1.85 | 0.056 | 1.36 | 0.99, 1.85 | 0.056 | 1.01 | 0.66, 1.56 | >0.9 | 0.96 | 0.63, 1.49 | 0.900 | 0.95 | 0.62, 1.46 | 0.800 |
| **Chronic Condition** |  |  |  |  |  |  |  |  |  |  |  |  |  |  |  |  |  |  |  |  |  |  |  |  |  |  |  |
| None | Ref | Ref | Ref | Ref | Ref | Ref | Ref | Ref | Ref | Ref | Ref | Ref | Ref | Ref | Ref | Ref | Ref | Ref | Ref | Ref | Ref | Ref | Ref | Ref | Ref | Ref | Ref |
| 1 - 2 | 0.61 | 0.43, 0.87 | **0.006** | 0.61 | 0.42, 0.87 | **0.006** | 0.61 | 0.43, 0.88 | **0.007** | 0.59 | 0.41, 0.84 | **0.003** | 0.58 | 0.40, 0.84 | **0.004** | 0.59 | 0.41, 0.84 | **0.004** | 0.79 | 0.47, 1.33 | 0.400 | 0.79 | 0.47, 1.33 | 0.400 | 0.78 | 0.46, 1.31 | 0.300 |
| >= 3 | 0.81 | 0.53, 1.25 | 0.300 | 0.78 | 0.50, 1.22 | 0.300 | 0.79 | 0.51, 1.23 | 0.300 | 0.80 | 0.51, 1.23 | 0.300 | 0.78 | 0.50, 1.21 | 0.300 | 0.78 | 0.50, 1.22 | 0.300 | 0.68 | 0.37, 1.26 | 0.200 | 0.64 | 0.34, 1.19 | 0.200 | 0.65 | 0.35, 1.21 | 0.200 |
| **Eyesight** |  |  |  |  |  |  |  |  |  |  |  |  |  |  |  |  |  |  |  |  |  |  |  |  |  |  |  |
| Excellent | Ref | Ref | Ref | Ref | Ref | Ref | Ref | Ref | Ref | Ref | Ref | Ref | Ref | Ref | Ref | Ref | Ref | Ref | Ref | Ref | Ref | Ref | Ref | Ref | Ref | Ref | Ref |
| Very Good | 0.88 | 0.48, 1.60 | 0.700 | 0.88 | 0.48, 1.60 | 0.700 | 0.86 | 0.47, 1.58 | 0.600 | 0.85 | 0.47, 1.55 | 0.600 | 0.85 | 0.46, 1.56 | 0.600 | 0.84 | 0.46, 1.55 | 0.600 | 0.68 | 0.25, 1.85 | 0.500 | 0.61 | 0.22, 1.68 | 0.300 | 0.61 | 0.22, 1.66 | 0.300 |
| Good | 0.95 | 0.54, 1.65 | 0.900 | 0.95 | 0.54, 1.66 | 0.900 | 0.94 | 0.54, 1.65 | 0.800 | 0.94 | 0.54, 1.64 | 0.800 | 0.95 | 0.54, 1.66 | 0.800 | 0.94 | 0.54, 1.65 | 0.800 | 0.74 | 0.29, 1.92 | 0.500 | 0.66 | 0.25, 1.71 | 0.400 | 0.65 | 0.25, 1.70 | 0.400 |
| Fair | 1.12 | 0.61, 2.03 | 0.700 | 1.15 | 0.63, 2.09 | 0.700 | 1.14 | 0.63, 2.09 | 0.700 | 1.11 | 0.61, 2.04 | 0.700 | 1.14 | 0.62, 2.09 | 0.700 | 1.14 | 0.62, 2.09 | 0.700 | 1.09 | 0.41, 2.87 | 0.900 | 1.05 | 0.40, 2.77 | >0.9 | 1.03 | 0.39, 2.73 | >0.9 |
| Poor | 1.18 | 0.59, 2.36 | 0.600 | 1.29 | 0.64, 2.60 | 0.500 | 1.29 | 0.64, 2.59 | 0.500 | 1.13 | 0.56, 2.28 | 0.700 | 1.24 | 0.61, 2.50 | 0.600 | 1.24 | 0.61, 2.50 | 0.600 | 1.53 | 0.55, 4.26 | 0.400 | 1.44 | 0.51, 4.03 | 0.500 | 1.42 | 0.51, 3.97 | 0.500 |
| Blind | - | - | - | - | - | - | - | - | - | - | - | - | - | - | - | - | - | - | - | - | - | - | - | - | - | - | - |
| **Hearing** |  |  |  |  |  |  |  |  |  |  |  |  |  |  |  |  |  |  |  |  |  |  |  |  |  |  |  |
| Excellent | Ref | Ref | Ref | Ref | Ref | Ref | Ref | Ref | Ref | Ref | Ref | Ref | Ref | Ref | Ref | Ref | Ref | Ref | Ref | Ref | Ref | Ref | Ref | Ref | Ref | Ref | Ref |
| Very Good | 0.81 | 0.57, 1.14 | 0.200 | 0.81 | 0.58, 1.15 | 0.200 | 0.82 | 0.58, 1.15 | 0.300 | 0.78 | 0.55, 1.10 | 0.200 | 0.78 | 0.55, 1.11 | 0.200 | 0.78 | 0.55, 1.11 | 0.200 | 0.85 | 0.51, 1.43 | 0.500 | 0.84 | 0.50, 1.41 | 0.500 | 0.84 | 0.50, 1.40 | 0.500 |
| Good | 1.07 | 0.78, 1.47 | 0.700 | 1.04 | 0.76, 1.43 | 0.800 | 1.04 | 0.76, 1.43 | 0.800 | 1.05 | 0.76, 1.44 | 0.800 | 1.03 | 0.75, 1.42 | 0.900 | 1.03 | 0.75, 1.42 | 0.900 | 1.05 | 0.66, 1.66 | 0.800 | 1.04 | 0.66, 1.64 | 0.900 | 1.04 | 0.65, 1.64 | 0.900 |
| Fair | 0.55 | 0.34, 0.89 | **0.014** | 0.53 | 0.33, 0.86 | **0.010** | 0.54 | 0.33, 0.87 | **0.011** | 0.52 | 0.32, 0.84 | **0.008** | 0.51 | 0.31, 0.82 | **0.006** | 0.51 | 0.31, 0.83 | **0.007** | 0.92 | 0.52, 1.62 | 0.800 | 0.96 | 0.54, 1.69 | 0.900 | 0.96 | 0.54, 1.69 | 0.900 |
| Poor | 0.78 | 0.35, 1.76 | 0.600 | 0.81 | 0.36, 1.84 | 0.600 | 0.83 | 0.37, 1.89 | 0.700 | 0.73 | 0.32, 1.66 | 0.500 | 0.77 | 0.34, 1.76 | 0.500 | 0.79 | 0.35, 1.81 | 0.600 | 0.66 | 0.20, 2.19 | 0.500 | 0.65 | 0.19, 2.17 | 0.500 | 0.67 | 0.20, 2.26 | 0.500 |

Each model further adjusted for Smoking, Alcohol Consumption, BMI, Diabetes, Depression, Brian Condition (stroke, psychiatric problems), Chronic Condition (high blood pressure, diabetes, cancer, lung disease, heart disease, and arthritis), Eyesight, and Hearing.
