# Supplementary material for: Associations of perceived neighborhood factors and Alzheimer’s disease polygenic score with cognition: Evidence from the Health and Retirement Study
Source: PLoS One. 2025 Nov 20;20(11):e0336403. doi: 10.1371/journal.pone.0336403 (PMC12633890; doi:10.1371/journal.pone.0336403)
Supplement: S5 Table — All models adjusted for Age, Sex, Education, Poverty Status, APOE E4 Status, Social Ladder, and Baseline Wave. (DOCX) [file pone.0336403.s005.docx]

**Supplemental Table 5.** Hazard Ratios from survival analysis using individual neighborhood factor as exposure, stratified by Ancestry: risk of incident cognitive impairment (CIND and dementia), CIND and dementia, relative to normal cognition and non-dementia in the US Health and Retirement Study (2008-2010 Waves).

|  | **Cognitive Impairment vs. Normal Cognition, European Ancestry (n=6,123)** | | | | | | | | | **CIND vs. Normal Cognition, European Ancestry (n=6,051)** | | | | | | | | | **Dementia vs. Non-dementia, European Ancestry (n=6,789)** | | | | | | | | |
| --- | --- | --- | --- | --- | --- | --- | --- | --- | --- | --- | --- | --- | --- | --- | --- | --- | --- | --- | --- | --- | --- | --- | --- | --- | --- | --- | --- |
|  | **Model 1** | | | **Model 2** | | | **Model 3** | | | **Model 1** | | | **Model 2** | | | **Model 3** | | | **Model 1** | | | **Model 2** | | | **Model 3** | | |
|  | **HR** | **95% CI** | **p-value** | **HR** | **95% CI** | **p-value** | **HR** | **95% CI** | **p-value** | **HR** | **95% CI** | **p-value** | **HR** | **95% CI** | **p-value** | **HR** | **95% CI** | **p-value** | **HR** | **95% CI** | **p-value** | **HR** | **95% CI** | **p-value** | **HR** | **95% CI** | **p-value** |
| **Neighborhood Safety** | 1.12 | 1.07, 1.18 | **<0.001** | 1.12 | 1.06, 1.18 | **<0.001** | 1.12 | 1.06, 1.18 | **<0.001** | 1.12 | 1.06, 1.18 | **<0.001** | 1.12 | 1.06, 1.18 | **<0.001** | 1.12 | 1.06, 1.18 | **<0.001** | 1.16 | 1.06, 1.28 | **0.002** | 1.16 | 1.05, 1.27 | **0.003** | 1.16 | 1.05, 1.27 | **0.003** |
| PGS-AD | - | - | **-** | 1.10 | 1.05, 1.16 | **<0.001** | 1.10 | 1.05, 1.15 | **<0.001** | - | - | **-** | 1.10 | 1.05, 1.16 | **<0.001** | 1.10 | 1.04, 1.15 | **<0.001** | - | - | **-** | 1.05 | 0.95, 1.15 | 0.300 | 1.05 | 0.95, 1.15 | 0.3 |
| Neighborhood Safety*PGS-AD | - | - | - | - | - | - | 0.96 | 0.91, 1.01 | 0.13 | - | - | - | - | - | - | 0.96 | 0.91, 1.01 | 0.15 | - | - | - | - | - | - | 0.97 | 0.88, 1.07 | 0.5 |
| **Neighborhood Trust** | 1.06 | 1.01, 1.12 | **0.029** | 1.07 | 1.01, 1.13 | **0.024** | 1.07 | 1.01, 1.13 | **0.024** | 1.06 | 1.00, 1.12 | **0.041** | 1.06 | 1.00, 1.12 | **0.036** | 1.06 | 1.00, 1.12 | **0.037** | 1.09 | 0.98, 1.20 | 0.100 | 1.08 | 0.98, 1.20 | 0.120 | 1.08 | 0.98, 1.20 | 0.130 |
| PGS-AD | - | - | **-** | 1.10 | 1.05, 1.16 | **<0.001** | 1.10 | 1.05, 1.16 | **<0.001** | - | - | **-** | 1.10 | 1.05, 1.16 | **<0.001** | 1.10 | 1.05, 1.16 | **<0.001** | - | - | **-** | 1.05 | 0.96, 1.16 | 0.300 | 1.05 | 0.95, 1.16 | 0.4 |
| Neighborhood Trust * PGS-AD | - | - | - | - | - | - | 0.99 | 0.94, 1.05 | 0.7 | - | - | - | - | - | - | 0.99 | 0.94, 1.05 | 0.8 | - | - | - | - | - | - | 0.98 | 0.89, 1.08 | 0.7 |
| **Neighborhood Friendly** | 1.07 | 1.01, 1.12 | **0.017** | 1.07 | 1.01, 1.12 | **0.015** | 1.07 | 1.01, 1.12 | **0.017** | 1.06 | 1.01, 1.12 | **0.031** | 1.06 | 1.01, 1.12 | **0.029** | 1.06 | 1.01, 1.12 | **0.032** | 1.09 | 0.99, 1.19 | 0.089 | 1.08 | 0.98, 1.19 | 0.110 | 1.08 | 0.98, 1.19 | 0.12 |
| PGS-AD | - | - | **-** | 1.10 | 1.05, 1.16 | **<0.001** | 1.10 | 1.05, 1.15 | **<0.001** | - | - | **-** | 1.10 | 1.05, 1.16 | **<0.001** | 1.10 | 1.05, 1.15 | **<0.001** | - | - | **-** | 1.05 | 0.96, 1.16 | 0.300 | 1.05 | 0.95, 1.15 | 0.3 |
| Neighborhood Friendly * PGS-AD | - | - | - | - | - | - | 0.97 | 0.93, 1.02 | 0.3 | - | - | - | - | - | - | 0.98 | 0.93, 1.03 | 0.4 | - | - | - | - | - | - | 0.97 | 0.88, 1.06 | 0.5 |
| **Neighborhood Vandalism** | 1.06 | 1.00, 1.11 | **0.040** | 1.06 | 1.00, 1.12 | **0.033** | 1.06 | 1.00, 1.12 | **0.033** | 1.05 | 1.00, 1.11 | 0.066 | 1.05 | 1.00, 1.11 | 0.057 | 1.05 | 1.00, 1.11 | 0.058 | 1.09 | 0.99, 1.21 | 0.068 | 1.09 | 0.99, 1.20 | 0.079 | 1.09 | 0.99, 1.20 | 0.081 |
| PGS-AD | - | - | **-** | 1.10 | 1.05, 1.16 | **<0.001** | 1.11 | 1.05, 1.16 | **<0.001** | - | - | **-** | 1.10 | 1.05, 1.16 | **<0.001** | 1.1 | 1.05, 1.16 | **<0.001** | - | - | **-** | 1.05 | 0.96, 1.16 | 0.300 | 1.05 | 0.95, 1.16 | 0.3 |
| Neighborhood Vandalism* PGS-AD | - | - | **-** | - | - | **-** | 1.02 | 0.97, 1.07 | 0.500 | - | - | **-** | - | - | **-** | 1.02 | 0.96, 1.07 | 0.6 | - | - | **-** | - | - | **-** | 0.98 | 0.90, 1.08 | 0.7 |
| **Neighborhood Belonging** | 1.06 | 1.01, 1.11 | **0.030** | 1.06 | 1.00, 1.11 | **0.034** | 1.06 | 1.00, 1.11 | **0.038** | 1.05 | 1.00, 1.11 | **0.049** | 1.05 | 1.00, 1.11 | 0.058 | 1.05 | 1.00, 1.11 | 0.066 | 1.1 | 1.00, 1.21 | **0.044** | 1.1 | 1.00, 1.21 | **0.050** | 1.10 | 1.00, 1.21 | 0.052 |
| PGS-AD | - | - | **-** | 1.10 | 1.05, 1.16 | **<0.001** | 1.10 | 1.05, 1.15 | **<0.001** | - | - | **-** | 1.10 | 1.05, 1.16 | **<0.001** | 1.10 | 1.04, 1.15 | **<0.001** | - | - | **-** | 1.05 | 0.95, 1.16 | 0.300 | 1.05 | 0.95, 1.16 | 0.300 |
| Neighborhood Belonging * PGS-AD | - | - | - | - | - | - | 0.96 | 0.91, 1.01 | 0.11 | - | - | - | - | - | - | 0.95 | 0.91, 1.01 | 0.082 | - | - | - | - | - | - | 0.99 | 0.89, 1.09 | 0.800 |
| **Neighborhood Cleanness** | 1.06 | 1.01, 1.12 | **0.024** | 1.06 | 1.01, 1.12 | **0.032** | 1.06 | 1.01, 1.12 | **0.032** | 1.06 | 1.00, 1.12 | **0.041** | 1.06 | 1.00, 1.11 | 0.055 | 1.05 | 1.00, 1.11 | 0.056 | 1.11 | 1.01, 1.22 | **0.038** | 1.10 | 1.00, 1.21 | 0.055 | 1.10 | 1.00, 1.21 | 0.057 |
| PGS-AD | - | - | **-** | 1.10 | 1.05, 1.16 | **<0.001** | 1.10 | 1.05, 1.16 | **<0.001** | - | - | **-** | 1.10 | 1.05, 1.16 | **<0.001** | 1.1 | 1.05, 1.16 | **<0.001** | - | - | **-** | 1.05 | 0.95, 1.16 | 0.300 | 1.05 | 0.95, 1.15 | 0.3 |
| Neighborhood Cleanness * PGS-AD | - | - | - | - | - | - | 1 | 0.95, 1.05 | >0.9 | - | - | - | - | - | - | 1 | 0.95, 1.06 | 0.9 | - | - | - | - | - | - | 0.98 | 0.89, 1.08 | 0.7 |
| **Neighborhood Vacant** | 1.04 | 0.99, 1.09 | 0.140 | 1.04 | 0.99, 1.09 | 0.200 | 1.04 | 0.98, 1.09 | 0.2 | 1.04 | 0.98, 1.09 | 0.200 | 1.04 | 0.98, 1.09 | 0.200 | 1.03 | 0.98, 1.09 | 0.2 | 1.1 | 1.00, 1.21 | 0.052 | 1.1 | 1.00, 1.21 | 0.059 | 1.10 | 1.00, 1.21 | 0.059 |
| PGS-AD | - | - | **-** | 1.10 | 1.05, 1.16 | **<0.001** | 1.11 | 1.06, 1.17 | **<0.001** | - | - | **-** | 1.10 | 1.05, 1.16 | **<0.001** | 1.11 | 1.06, 1.17 | **<0.001** | - | - | **-** | 1.05 | 0.95, 1.16 | 0.300 | 1.05 | 0.95, 1.16 | 0.3 |
| Neighborhood Vacant * PGS-AD | - | - | - | - | - | - | 1.06 | 1.01, 1.12 | **0.023** | - | - | - | - | - | - | 1.06 | 1.01, 1.12 | **0.022** | - | - | - | - | - | - | 1.01 | 0.92, 1.12 | 0.8 |
|  | **Cognitive Impairment vs. Normal Cognition, African Ancestry (n=703)** | | | | | | | | | **CIND vs. Normal Cognition, African Ancestry (n=695)** | | | | | | | | | **Dementia vs. Non-dementia, African Ancestry (n=971)** | | | | | | | | |
|  | **Model 1** | | | **Model 2** | | | **Model 3** | | | **Model 1** | | | **Model 2** | | | **Model 3** | | | **Model 1** | | | **Model 2** | | | **Model 3** | | |
|  | **HR** | **95% CI** | **p-value** | **HR** | **95% CI** | **p-value** | **HR** | **95% CI** | **p-value** | **HR** | **95% CI** | **p-value** | **HR** | **95% CI** | **p-value** | **HR** | **95% CI** | **p-value** | **HR** | **95% CI** | **p-value** | **HR** | **95% CI** | **p-value** | **HR** | **95% CI** | **p-value** |
| **Neighborhood Safety** | 1.03 | 0.92, 1.14 | 0.600 | 1.03 | 0.93, 1.15 | 0.600 | 1.04 | 0.93, 1.16 | 0.5 | 1.03 | 0.92, 1.14 | 0.600 | 1.04 | 0.93, 1.15 | 0.500 | 1.04 | 0.93, 1.16 | 0.500 | 1.07 | 0.93, 1.22 | 0.400 | 1.07 | 0.93, 1.24 | 0.300 | 1.08 | 0.94, 1.25 | 0.300 |
| PGS-AD | - | - | - | 1.13 | 0.95, 1.35 | 0.200 | 1.15 | 0.96, 1.37 | 0.13 | - | - | - | 1.11 | 0.93, 1.33 | 0.200 | 1.12 | 0.94, 1.34 | 0.2 | - | - | - | 1.10 | 0.86, 1.39 | 0.500 | 1.14 | 0.89, 1.46 | 0.3 |
| Neighborhood Safety*PGS-AD | - | - | - | - | - | - | 0.95 | 0.84, 1.07 | 0.4 | - | - | - | - | - | - | 0.96 | 0.85, 1.08 | 0.5 | - | - | - | - | - | - | 0.87 | 0.74, 1.03 | 0.11 |
| **Neighborhood Trust** | 1.01 | 0.91, 1.13 | 0.900 | 0.99 | 0.89, 1.11 | 0.900 | 1.01 | 0.91, 1.13 | 0.800 | 0.99 | 0.89, 1.11 | >0.9 | 0.97 | 0.87, 1.09 | 0.600 | 1.00 | 0.89, 1.12 | >0.9 | 1.06 | 0.93, 1.22 | 0.400 | 1.06 | 0.92, 1.22 | 0.400 | 1.07 | 0.93, 1.23 | 0.300 |
| PGS-AD | - | - | - | 1.14 | 0.95, 1.35 | 0.200 | 1.18 | 0.99, 1.42 | 0.065 | - | - | - | 1.12 | 0.94, 1.33 | 0.200 | 1.18 | 0.98, 1.41 | 0.073 | - | - | - | 1.10 | 0.86, 1.39 | 0.400 | 1.12 | 0.87, 1.44 | 0.4 |
| Neighborhood Trust * PGS-AD | - | - | - | - | - | - | 0.88 | 0.77, 1.01 | 0.064 | - | - | - | - | - | - | 0.86 | 0.75, 0.98 | **0.025** | - | - | - | - | - | - | 0.95 | 0.81, 1.13 | 0.6 |
| **Neighborhood Friendly** | 1.11 | 1.00, 1.23 | 0.051 | 1.12 | 1.00, 1.24 | **0.041** | 1.12 | 1.00, 1.24 | **0.046** | 1.11 | 1.00, 1.24 | **0.050** | 1.12 | 1.01, 1.25 | **0.039** | 1.12 | 1.00, 1.25 | **0.048** | 1.01 | 0.88, 1.17 | 0.800 | 1.02 | 0.89, 1.17 | 0.800 | 1.08 | 0.94, 1.25 | 0.3 |
| PGS-AD | - | - | - | 1.13 | 0.95, 1.35 | 0.200 | 1.13 | 0.94, 1.35 | 0.2 | - | - | - | 1.11 | 0.93, 1.33 | 0.200 | 1.11 | 0.93, 1.33 | 0.3 | - | - | - | 1.09 | 0.86, 1.39 | 0.500 | 1.14 | 0.89, 1.46 | 0.3 |
| Neighborhood Friendly * PGS-AD | - | - | - | - | - | - | 1.00 | 0.87, 1.15 | >0.9 | - | - | - | - | - | - | 1.01 | 0.88, 1.17 | 0.8 | - | - | - | - | - | - | 0.87 | 0.74, 1.03 | 0.11 |
| **Neighborhood Vandalism** | 1.02 | 0.92, 1.13 | 0.700 | 1.02 | 0.92, 1.13 | 0.700 | 1.03 | 0.93, 1.15 | 0.5 | 1.01 | 0.91, 1.12 | 0.800 | 1.01 | 0.91, 1.13 | 0.800 | 1.02 | 0.92, 1.14 | 0.7 | 0.99 | 0.87, 1.13 | 0.900 | 0.99 | 0.87, 1.13 | >0.9 | 1.00 | 0.87, 1.14 | >0.9 |
| PGS-AD | - | - | **-** | 1.13 | 0.95, 1.35 | 0.200 | 1.15 | 0.96, 1.37 | 0.12 | - | - | **-** | 1.11 | 0.94, 1.33 | 0.200 | 1.12 | 0.94, 1.34 | 0.2 | - | - | **-** | 1.09 | 0.86, 1.39 | 0.500 | 1.11 | 0.87, 1.41 | 0.4 |
| Neighborhood Vandalism* PGS-AD | - | - | - | - | - | - | 0.93 | 0.82, 1.04 | 0.2 | - | - | - | - | - | - | 0.95 | 0.84, 1.07 | 0.400 | - | - | - | - | - | - | 0.93 | 0.79, 1.09 | 0.4 |
| **Neighborhood Belonging** | 1.08 | 0.97, 1.19 | 0.200 | 1.07 | 0.97, 1.18 | 0.200 | 1.07 | 0.97, 1.19 | 0.2 | 1.08 | 0.97, 1.19 | 0.200 | 1.07 | 0.97, 1.19 | 0.200 | 1.07 | 0.97, 1.19 | 0.200 | 0.97 | 0.84, 1.11 | 0.600 | 0.97 | 0.84, 1.11 | 0.600 | 0.97 | 0.84, 1.12 | 0.7 |
| PGS-AD | - | - | **-** | 1.13 | 0.95, 1.35 | 0.200 | 1.13 | 0.95, 1.35 | 0.2 | - | - | **-** | 1.11 | 0.93, 1.33 | 0.200 | 1.11 | 0.93, 1.33 | 0.200 | - | - | **-** | 1.09 | 0.86, 1.39 | 0.500 | 1.10 | 0.87, 1.40 | 0.4 |
| Neighborhood Belonging * PGS-AD | - | - | - | - | - | - | 0.99 | 0.88, 1.11 | 0.9 | - | - | - | - | - | - | 0.99 | 0.89, 1.12 | >0.9 | - | - | - | - | - | - | 0.90 | 0.77, 1.05 | 0.2 |
| **Neighborhood Cleanness** | 1.02 | 0.91, 1.13 | 0.800 | 1.02 | 0.91, 1.13 | 0.700 | 1.02 | 0.92, 1.14 | 0.7 | 1.01 | 0.90, 1.12 | 0.900 | 1.01 | 0.90, 1.12 | 0.900 | 1.01 | 0.91, 1.13 | 0.8 | 1.01 | 0.88, 1.16 | 0.900 | 1.02 | 0.89, 1.17 | 0.800 | 1.03 | 0.90, 1.18 | 0.7 |
| PGS-AD | - | - | - | 1.13 | 0.95, 1.35 | 0.200 | 1.14 | 0.95, 1.36 | 0.2 | - | - | - | 1.11 | 0.94, 1.33 | 0.200 | 1.12 | 0.94, 1.35 | 0.2 | - | - | - | 1.09 | 0.86, 1.39 | 0.500 | 1.14 | 0.89, 1.46 | 0.3 |
| Neighborhood Cleanness * PGS-AD | - | - | - | - | - | - | 0.98 | 0.86, 1.12 | 0.8 | - | - | - | - | - | - | 0.97 | 0.85, 1.11 | 0.7 | - | - | - | - | - | - | 0.9 | 0.77, 1.05 | 0.2 |
| **Neighborhood Vacant** | 0.95 | 0.85, 1.06 | 0.400 | 0.95 | 0.86, 1.06 | 0.400 | 0.96 | 0.86, 1.08 | 0.500 | 0.95 | 0.86, 1.06 | 0.400 | 0.96 | 0.86, 1.07 | 0.500 | 0.97 | 0.87, 1.08 | 0.6 | 0.99 | 0.86, 1.15 | >0.9 | 1.00 | 0.86, 1.15 | >0.9 | 0.99 | 0.85, 1.14 | 0.9 |
| PGS-AD | - | - | **-** | 1.13 | 0.95, 1.35 | 0.200 | 1.15 | 0.96, 1.37 | 0.130 | - | - | **-** | 1.11 | 0.93, 1.33 | 0.200 | 1.12 | 0.94, 1.34 | 0.2 | - | - | **-** | 1.09 | 0.86, 1.39 | 0.500 | 1.07 | 0.84, 1.36 | 0.6 |
| Neighborhood Vacant * PGS-AD | - | - | - | - | - | - | 0.94 | 0.83, 1.06 | 0.300 | - | - | - | - | - | - | 0.96 | 0.85, 1.08 | 0.5 | - | - | - | - | - | - | 1.12 | 0.95, 1.32 | 0.2 |

All Models adjusted for Age, Sex, Education, Poverty Status, APOE E4 Status, Social Ladder, and Baseline Wave.
