# Supplementary material for: Associations of perceived neighborhood factors and Alzheimer’s disease polygenic score with cognition: Evidence from the Health and Retirement Study
Source: PLoS One. 2025 Nov 20;20(11):e0336403. doi: 10.1371/journal.pone.0336403 (PMC12633890; doi:10.1371/journal.pone.0336403)
Supplement: S6 Table — (DOCX) [file pone.0336403.s006.docx]

**Supplemental Table 6.** Hazard Ratios from survival analysis comparing binary Neighborhood disadvantage index with additional covariates, stratified by Ancestry: risk of incident cognitive impairment (CIND and dementia), CIND and dementia in the US Health and Retirement Study (2008-2010 Waves).

|  | **Cognitive Impairment vs. Normal Cognition, European Ancestry (n=6,031)** | | | | | | | | | **CIND vs. Normal Cognition, European Ancestry (n=5,962)** | | | | | | | | | **Dementia vs. Non-dementia, European Ancestry (n=6,685)** | | | | | | | | |
| --- | --- | --- | --- | --- | --- | --- | --- | --- | --- | --- | --- | --- | --- | --- | --- | --- | --- | --- | --- | --- | --- | --- | --- | --- | --- | --- | --- |
|  | **Model 1** | | | **Model 2** | | | **Model 3** | | | **Model 1** | | | **Model 2** | | | **Model 3** | | | **Model 1** | | | **Model 2** | | | **Model 3** | | |
|  | **HR** | **95% CI** | **p-value** | **HR** | **95% CI** | **p-value** | **HR** | **95% CI** | **p-value** | **HR** | **95% CI** | **p-value** | **HR** | **95% CI** | **p-value** | **HR** | **95% CI** | **p-value** | **HR** | **95% CI** | **p-value** | **HR** | **95% CI** | **p-value** | **HR** | **95% CI** | **p-value** |
| **Neighborhood disadvantage index** |  |  |  |  |  |  |  |  |  |  |  |  |  |  |  |  |  |  |  |  |  |  |  |  |  |  |  |
| The least disadvantaged neighborhoods (<=0) | Ref | Ref | Ref | Ref | Ref | Ref | Ref | Ref | Ref | Ref | Ref | Ref | Ref | Ref | Ref | Ref | Ref | Ref | Ref | Ref | Ref | Ref | Ref | Ref | Ref | Ref | Ref |
| The most disadvantaged neighborhoods (>0) | 1.14 | 1.03, 1.26 | **0.014** | 1.14 | 1.03, 1.27 | **0.011** | 1.16 | 1.03, 1.30 | **0.017** | 1.13 | 1.02, 1.26 | **0.024** | 1.13 | 1.02, 1.26 | **0.020** | 1.15 | 1.02, 1.29 | **0.026** | 1.29 | 1.05, 1.58 | **0.013** | 1.29 | 1.05, 1.58 | **0.014** | 1.28 | 1.01, 1.61 | **0.038** |
| **Age** | 1.08 | 1.07, 1.08 | **<0.001** | 1.08 | 1.07, 1.08 | **<0.001** | 1.08 | 1.07, 1.08 | **<0.001** | 1.08 | 1.07, 1.08 | **<0.001** | 1.08 | 1.07, 1.08 | **<0.001** | 1.08 | 1.07, 1.08 | **<0.001** | 1.11 | 1.10, 1.13 | **<0.001** | 1.11 | 1.10, 1.13 | **<0.001** | 1.11 | 1.10, 1.13 | **<0.001** |
| **Sex** |  |  |  |  |  |  |  |  |  |  |  |  |  |  |  |  |  |  |  |  |  |  |  |  |  |  |  |
| Female | Ref | Ref | Ref | Ref | Ref | Ref | Ref | Ref | Ref | Ref | Ref | Ref | Ref | Ref | Ref | Ref | Ref | Ref | Ref | Ref | Ref | Ref | Ref | Ref | Ref | Ref | Ref |
| Male | 1.26 | 1.13, 1.39 | **<0.001** | 1.25 | 1.13, 1.39 | **<0.001** | 1.25 | 1.13, 1.39 | **<0.001** | 1.26 | 1.13, 1.40 | **<0.001** | 1.26 | 1.13, 1.40 | **<0.001** | 1.26 | 1.13, 1.40 | **<0.001** | 1.14 | 0.92, 1.41 | 0.200 | 1.15 | 0.93, 1.42 | 0.200 | 1.15 | 0.93, 1.42 | 0.200 |
| **Education** |  |  |  |  |  |  |  |  |  |  |  |  |  |  |  |  |  |  |  |  |  |  |  |  |  |  |  |
| Above High School/GED | Ref | Ref | Ref | Ref | Ref | Ref | Ref | Ref | Ref | Ref | Ref | Ref | Ref | Ref | Ref | Ref | Ref | Ref | Ref | Ref | Ref | Ref | Ref | Ref | Ref | Ref | Ref |
| High School/GED | 1.58 | 1.41, 1.78 | **<0.001** | 1.59 | 1.41, 1.79 | **<0.001** | 1.59 | 1.41, 1.79 | **<0.001** | 1.61 | 1.43, 1.82 | **<0.001** | 1.62 | 1.43, 1.82 | **<0.001** | 1.62 | 1.43, 1.82 | **<0.001** | 1.66 | 1.28, 2.15 | **<0.001** | 1.67 | 1.29, 2.17 | **<0.001** | 1.67 | 1.29, 2.17 | **<0.001** |
| Less than High School/GED | 2.78 | 2.35, 3.30 | **<0.001** | 2.78 | 2.35, 3.30 | **<0.001** | 2.78 | 2.34, 3.30 | **<0.001** | 2.83 | 2.38, 3.38 | **<0.001** | 2.84 | 2.38, 3.38 | **<0.001** | 2.84 | 2.38, 3.38 | **<0.001** | 4.01 | 2.95, 5.46 | **<0.001** | 4.06 | 2.98, 5.52 | **<0.001** | 4.06 | 2.98, 5.53 | **<0.001** |
| **Poverty Status (Below)** |  |  |  |  |  |  |  |  |  |  |  |  |  |  |  |  |  |  |  |  |  |  |  |  |  |  |  |
| Above Poverty threshold | Ref | Ref | Ref | Ref | Ref | Ref | Ref | Ref | Ref | Ref | Ref | Ref | Ref | Ref | Ref | Ref | Ref | Ref | Ref | Ref | Ref | Ref | Ref | Ref | Ref | Ref | Ref |
| Below Poverty threshold | 1.15 | 0.89, 1.49 | 0.300 | 1.15 | 0.88, 1.49 | 0.300 | 1.15 | 0.88, 1.49 | 0.300 | 1.16 | 0.89, 1.51 | 0.300 | 1.16 | 0.89, 1.51 | **0.300** | 1.16 | 0.89, 1.51 | 0.300 | 1.79 | 1.20, 2.66 | **0.004** | 1.83 | 1.23, 2.73 | **0.003** | 1.83 | 1.23, 2.73 | **0.003** |
| **APOE E4 status (No copy)** |  |  |  |  |  |  |  |  |  |  |  |  |  |  |  |  |  |  |  |  |  |  |  |  |  |  |  |
| No copies of e4 | Ref | Ref | Ref | Ref | Ref | Ref | Ref | Ref | Ref | Ref | Ref | Ref | Ref | Ref | Ref | Ref | Ref | Ref | Ref | Ref | Ref | Ref | Ref | Ref | Ref | Ref | Ref |
| Any copies of e4 | 1.44 | 1.30, 1.60 | **<0.001** | 1.44 | 1.29, 1.60 | **<0.001** | 1.43 | 1.29, 1.59 | **<0.001** | 1.45 | 1.30, 1.61 | **<0.001** | 1.44 | 1.29, 1.61 | **<0.001** | 1.44 | 1.29, 1.61 | **<0.001** | 2.06 | 1.70, 2.51 | **<0.001** | 2.05 | 1.69, 2.50 | **<0.001** | 2.05 | 1.69, 2.50 | **<0.001** |
| **Social Ladder** | 0.96 | 0.93, 0.99 | **0.010** | 0.96 | 0.93, 0.99 | **0.001** | 0.96 | 0.93, 0.99 | **0.010** | 0.96 | 0.93, 0.99 | **0.007** | 0.96 | 0.93, 0.99 | **0.001** | 0.96 | 0.93, 0.99 | **0.007** | 1.02 | 0.95, 1.08 | 0.6 | 1.02 | 0.96, 1.08 | 0.6 | 1.02 | 0.96, 1.08 | 0.6 |
| **Baseline wave** |  |  |  |  |  |  |  |  |  |  |  |  |  |  |  |  |  |  |  |  |  |  |  |  |  |  |  |
| Wave 1 (2008) | Ref | Ref | Ref | Ref | Ref | Ref | Ref | Ref | Ref | Ref | Ref | Ref | Ref | Ref | Ref | Ref | Ref | Ref | Ref | Ref | Ref | Ref | Ref | Ref | Ref | Ref | Ref |
| Wave 2 (2010) | 0.86 | 0.78, 0.95 | **0.002** | 0.87 | 0.79, 0.95 | **0.003** | 0.86 | 0.79, 0.95 | **0.003** | 0.86 | 0.79, 0.95 | **0.003** | 0.87 | 0.79, 0.96 | **0.004** | 0.87 | 0.79, 0.96 | **0.004** | 0.81 | 0.67, 0.98 | **0.029** | 0.81 | 0.67, 0.98 | **0.029** | 0.81 | 0.67, 0.98 | **0.030** |
| **PGS-AD** |  |  |  |  |  |  |  |  |  |  |  |  |  |  |  |  |  |  |  |  |  |  |  |  |  |  |  |
| Below 75% | - | - | - | Ref | Ref | Ref | Ref | Ref | Ref | - | - | - | Ref | Ref | Ref | Ref | Ref | Ref | - | - | - | Ref | Ref | Ref | Ref | Ref | Ref |
| Above 75% | - | - | - | 1.12 | 1.01, 1.26 | **0.039** | 1.14 | 1.00, 1.30 | 0.056 | - | - | - | 1.12 | 1.00, 1.26 | **0.048** | 1.14 | 0.99, 1.31 | 0.066 | - | - | - | 1.15 | 0.93, 1.43 | 0.2 | 1.14 | 0.87, 1.49 | 0.300 |
| **Neighborhood* PGS-AD** | - | - | - | - | - | - | 0.96 | 0.76, 1.21 | 0.7 | - | - | - | - | - | - | 0.96 | 0.75, 1.22 | 0.7 | - | - | - | - | - | - | 1.03 | 0.65, 1.62 | 0.9 |
| RERI: The most disadvantaged neighborhoods*PGS-AD Above 75% | - | - | - | - | - | - | 0.14 | -0.19, 0.46 |  | - | - | - | - | - | - | -0.16 | -0.18, 0.49 |  | - | - | - | - | - | - | -0.68 | -1.29, 0.08 |  |
| **Smoking status** |  |  |  |  |  |  |  |  |  |  |  |  |  |  |  |  |  |  |  |  |  |  |  |  |  |  |  |
| Never Smoker | Ref | Ref | Ref | Ref | Ref | Ref | Ref | Ref | Ref | Ref | Ref | Ref | Ref | Ref | Ref | Ref | Ref | Ref | Ref | Ref | Ref | Ref | Ref | Ref | Ref | Ref | Ref |
| Current Smoker | 1.23 | 1.04, 1.47 | **0.017** | 1.24 | 1.04, 1.47 | **0.015** | 1.24 | 1.04, 1.47 | **0.015** | 1.24 | 1.04, 1.48 | **0.016** | 1.25 | 1.05, 1.48 | **0.014** | 1.24 | 1.04, 1.48 | **0.014** | 1.00 | 0.68, 1.47 | >0.9 | 1.00 | 0.68, 1.46 | >0.9 | 1.00 | 0.68, 1.46 | >0.9 |
| Former Smoke | 1.07 | 0.96, 1.18 | 0.200 | 1.06 | 0.96, 1.18 | 0.2 | 1.06 | 0.96, 1.18 | 0.2 | 1.06 | 0.96, 1.18 | 0.3 | 1.06 | 0.95, 1.18 | 0.3 | 1.06 | 0.95, 1.18 | 0.3 | 0.99 | 0.81, 1.21 | >0.9 | 0.99 | 0.81, 1.21 | >0.9 | 0.99 | 0.81, 1.21 | >0.9 |
| **Drinking (# drinks/day when drinks)** | 1.01 | 0.97, 1.05 | 0.700 | 1.01 | 0.97, 1.05 | 0.7 | 1.01 | 0.97, 1.05 | 0.7 | 1.01 | 0.97, 1.05 | 0.7 | 1.01 | 0.97, 1.05 | 0.6 | 1.01 | 0.97, 1.05 | 0.6 | 1.02 | 0.93, 1.12 | 0.7 | 1.02 | 0.93, 1.12 | 0.7 | 1.02 | 0.93, 1.12 | 0.7 |
| **Depression** | 1.08 | 1.05, 1.11 | **<0.001** | 1.08 | 1.05, 1.11 | **<0.001** | 1.08 | 1.05, 1.11 | **<0.001** | 1.08 | 1.05, 1.11 | **<0.001** | 1.08 | 1.05, 1.11 | **<0.001** | 1.08 | 1.05, 1.11 | **<0.001** | 1.08 | 1.03, 1.14 | **0.004** | 1.08 | 1.03, 1.14 | **0.004** | 1.08 | 1.03, 1.14 | **0.004** |
| **BMI** | 1.0 | 0.99, 1.00 | 0.300 | 1.00 | 0.99, 1.01 | 0.400 | 1.00 | 0.99, 1.01 | 0.4 | 1.00 | 0.99, 1.01 | 0.4 | 1.00 | 0.99, 1.01 | 0.5 | 1.00 | 0.99, 1.01 | 0.5 | 0.97 | 0.95, 0.99 | **0.005** | 0.97 | 0.95, 0.99 | **0.006** | 0.97 | 0.95, 0.99 | **0.006** |
| **Ever have Diabetes** |  |  |  |  |  |  |  |  |  |  |  |  |  |  |  |  |  |  |  |  |  |  |  |  |  |  |  |
| No | Ref | Ref | Ref | Ref | Ref | Ref | Ref | Ref | Ref | Ref | Ref | Ref | Ref | Ref | Ref | Ref | Ref | Ref | Ref | Ref | Ref | Ref | Ref | Ref | Ref | Ref | Ref |
| Yes | 1.17 | 1.01, 1.34 | **0.031** | 1.15 | 1.00, 1.32 | **0.050** | 1.15 | 1.00, 1.32 | 0.051 | 1.19 | 1.03, 1.37 | **0.017** | 1.17 | 1.02, 1.35 | **0.030** | 1.17 | 1.02, 1.35 | **0.030** | 1.16 | 0.87, 1.53 | 0.3 | 1.15 | 0.87, 1.53 | 0.3 | 1.15 | 0.87, 1.53 | 0.3 |
| **Brain Condition** |  |  |  |  |  |  |  |  |  |  |  |  |  |  |  |  |  |  |  |  |  |  |  |  |  |  |  |
| No | Ref | Ref | Ref | Ref | Ref | Ref | Ref | Ref | Ref | Ref | Ref | Ref | Ref | Ref | Ref | Ref | Ref | Ref | Ref | Ref | Ref | Ref | Ref | Ref | Ref | Ref | Ref |
| Yes | 1.16 | 1.03, 1.31 | **0.014** | 1.16 | 1.03, 1.30 | **0.017** | 1.16 | 1.03, 1.30 | **0.017** | 1.15 | 1.02, 1.30 | **0.027** | 1.14 | 1.01, 1.29 | **0.032** | 1.14 | 1.01, 1.29 | **0.032** | 1.49 | 1.19, 1.87 | **<0.001** | 1.50 | 1.19, 1.87 | **<0.001** | 1.50 | 1.19, 1.87 | **<0.001** |
| **Chronic Condition** |  |  |  |  |  |  |  |  |  |  |  |  |  |  |  |  |  |  |  |  |  |  |  |  |  |  |  |
| None | Ref | Ref | Ref | Ref | Ref | Ref | Ref | Ref | Ref | Ref | Ref | Ref | Ref | Ref | Ref | Ref | Ref | Ref | Ref | Ref | Ref | Ref | Ref | Ref | Ref | Ref | Ref |
| 1 - 2 | 0.96 | 0.82, 1.12 | 0.6 | 0.96 | 0.82, 1.13 | 0.6 | 0.96 | 0.82, 1.13 | 0.6 | 0.97 | 0.83, 1.14 | 0.7 | 0.98 | 0.83, 1.15 | 0.8 | 0.98 | 0.83, 1.15 | 0.8 | 0.74 | 0.55, 1.01 | 0.057 | 0.74 | 0.55, 1.01 | 0.059 | 0.74 | 0.55, 1.01 | 0.059 |
| >= 3 | 1.02 | 0.85, 1.23 | 0.8 | 1.04 | 0.87, 1.25 | 0.7 | 1.04 | 0.87, 1.25 | 0.7 | 1.04 | 0.86, 1.25 | 0.7 | 1.05 | 0.87, 1.27 | 0.6 | 1.06 | 0.87, 1.27 | 0.6 | 0.63 | 0.44, 0.90 | **0.011** | 0.63 | 0.44, 0.90 | **0.011** | 0.63 | 0.44, 0.90 | **0.011** |
| **Eyesight** |  |  |  |  |  |  |  |  |  |  |  |  |  |  |  |  |  |  |  |  |  |  |  |  |  |  |  |
| Excellent | Ref | Ref | Ref | Ref | Ref | Ref | Ref | Ref | Ref | Ref | Ref | Ref | Ref | Ref | Ref | Ref | Ref | Ref | Ref | Ref | Ref | Ref | Ref | Ref | Ref | Ref | Ref |
| Very Good | 0.94 | 0.78, 1.13 | 0.5 | 0.95 | 0.79, 1.14 | 0.6 | 0.95 | 0.79, 1.14 | 0.6 | 0.95 | 0.79, 1.15 | 0.6 | 0.96 | 0.79, 1.16 | 0.6 | 0.96 | 0.79, 1.16 | 0.7 | 0.66 | 0.46, 0.96 | **0.031** | 0.67 | 0.46, 0.97 | **0.032** | 0.67 | 0.46, 0.97 | **0.032** |
| Good | 1.02 | 0.85, 1.22 | 0.9 | 1.02 | 0.85, 1.22 | 0.9 | 1.02 | 0.85, 1.22 | 0.8 | 1.02 | 0.85, 1.23 | 0.8 | 1.02 | 0.85, 1.23 | 0.8 | 1.02 | 0.85, 1.23 | 0.8 | 0.87 | 0.61, 1.23 | 0.4 | 0.87 | 0.61, 1.23 | 0.4 | 0.87 | 0.61, 1.23 | 0.4 |
| Fair | 1.31 | 1.06, 1.61 | **0.012** | 1.30 | 1.06, 1.60 | **0.014** | 1.30 | 1.06, 1.60 | **0.014** | 1.31 | 1.06, 1.62 | **0.012** | 1.31 | 1.06, 1.62 | **0.013** | 1.31 | 1.06, 1.62 | **0.013** | 0.93 | 0.62, 1.39 | 0.7 | 0.91 | 0.61, 1.35 | 0.6 | 0.91 | 0.61, 1.35 | 0.6 |
| Poor | 1.06 | 0.78, 1.43 | 0.7 | 1.04 | 0.77, 1.41 | 0.8 | 1.04 | 0.77, 1.41 | 0.8 | 1.06 | 0.78, 1.45 | 0.7 | 1.04 | 0.76, 1.42 | 0.8 | 1.05 | 0.77, 1.42 | 0.8 | 1.21 | 0.75, 1.97 | 0.4 | 1.17 | 0.72, 1.90 | 0.5 | 1.17 | 0.72, 1.90 | 0.5 |
| Blind | 0.77 | 0.24, 2.43 | 0.7 | 0.72 | 0.23, 2.27 | 0.6 | 0.72 | 0.23, 2.27 | 0.6 | 0.59 | 0.15, 2.41 | 0.5 | 0.55 | 0.14, 2.24 | 0.4 | 0.55 | 0.14, 2.24 | 0.4 | 1.49 | 0.35, 6.28 | 0.6 | 1.41 | 0.33, 5.97 | 0.6 | 1.41 | 0.33, 5.97 | 0.6 |
| **Hearing** |  |  |  |  |  |  |  |  |  |  |  |  |  |  |  |  |  |  |  |  |  |  |  |  |  |  |  |
| Excellent | Ref | Ref | Ref | Ref | Ref | Ref | Ref | Ref | Ref | Ref | Ref | Ref | Ref | Ref | Ref | Ref | Ref | Ref | Ref | Ref | Ref | Ref | Ref | Ref | Ref | Ref | Ref |
| Very Good | 0.94 | 0.80, 1.10 | 0.4 | 0.94 | 0.80, 1.10 | 0.4 | 0.94 | 0.80, 1.10 | 0.4 | 0.93 | 0.79, 1.09 | 0.4 | 0.93 | 0.79, 1.09 | 0.4 | 0.93 | 0.79, 1.09 | 0.4 | 1.09 | 0.78, 1.53 | 0.6 | 1.11 | 0.79, 1.54 | 0.6 | 1.11 | 0.79, 1.55 | 0.6 |
| Good | 1.04 | 0.90, 1.22 | 0.6 | 1.04 | 0.89, 1.21 | 0.6 | 1.04 | 0.89, 1.21 | 0.6 | 1.04 | 0.89, 1.22 | 0.6 | 1.04 | 0.89, 1.21 | 0.6 | 1.04 | 0.89, 1.21 | 0.6 | 1.08 | 0.79, 1.49 | 0.6 | 1.09 | 0.79, 1.49 | 0.6 | 1.09 | 0.79, 1.49 | 0.6 |
| Fair | 1.24 | 1.04, 1.48 | **0.016** | 1.22 | 1.03, 1.46 | 0.024 | 1.22 | 1.03, 1.46 | **0.024** | 1.24 | 1.04, 1.48 | **0.019** | 1.22 | 1.02, 1.46 | **0.027** | 1.22 | 1.02, 1.46 | **0.028** | 1.30 | 0.92, 1.85 | 0.14 | 1.32 | 0.93, 1.88 | 0.12 | 1.32 | 0.93, 1.88 | 0.12 |
| Poor | 1.32 | 1.04, 1.68 | **0.021** | 1.30 | 1.02, 1.64 | 0.032 | 1.29 | 1.02, 1.64 | **0.034** | 1.34 | 1.05, 1.70 | **0.018** | 1.31 | 1.03, 1.67 | **0.028** | 1.31 | 1.03, 1.67 | **0.029** | 1.13 | 0.73, 1.77 | 0.6 | 1.14 | 0.73, 1.79 | 0.6 | 1.14 | 0.73, 1.79 | 0.6 |
|  | **Cognitive Impairment vs. Normal Cognition, African Ancestry (n=696)** | | | | | | | | | **CIND vs. Normal Cognition, African Ancestry (n=688)** | | | | | | | | | **Dementia vs. Non-dementia, African Ancestry (n=960)** | | | | | | | | |
|  | **Model 1** | | | **Model 2** | | | **Model 3** | | | **Model 1** | | | **Model 2** | | | **Model 3** | | | **Model 1** | | | **Model 2** | | | **Model 3** | | |
|  | **HR** | **95% CI** | **p-value** | **HR** | **95% CI** | **p-value** | **HR** | **95% CI** | **p-value** | **HR** | **95% CI** | **p-value** | **HR** | **95% CI** | **p-value** | **HR** | **95% CI** | **p-value** | **HR** | **95% CI** | **p-value** | **HR** | **95% CI** | **p-value** | **HR** | **95% CI** | **p-value** |
| **Neighborhood disadvantage index** |  |  |  |  |  |  |  |  |  |  |  |  |  |  |  |  |  |  |  |  |  |  |  |  |  |  |  |
| The least disadvantaged neighborhoods (<=0) | Ref | Ref | Ref | Ref | Ref | Ref | Ref | Ref | Ref | Ref | Ref | Ref | Ref | Ref | Ref | Ref | Ref | Ref | Ref | Ref | Ref | Ref | Ref | Ref | Ref | Ref | Ref |
| The most disadvantaged neighborhoods (>0) | 1.00 | 0.79, 1.27 | >0.9 | 0.97 | 0.76, 1.24 | 0.80 | 1.09 | 0.82, 1.45 | 0.6 | 1.0 | 0.78, 1.27 | >0.9 | 0.97 | 0.76, 1.24 | 0.8 | 1.06 | 0.80, 1.42 | 0.7 | 1.26 | 0.91, 1.76 | 0.2 | 1.27 | 0.91, 1.77 | 0.2 | 1.30 | 0.89, 1.91 | 0.2 |
| **Age** | 1.06 | 1.04, 1.07 | **<0.001** | 1.06 | 1.04, 1.08 | **<0.001** | 1.06 | 1.04, 1.08 | **<0.001** | 1.06 | 1.04, 1.08 | **<0.001** | 1.06 | 1.05, 1.08 | **<0.001** | 1.06 | 1.05, 1.08 | **<0.001** | 1.10 | 1.07, 1.12 | **<0.001** | 1.10 | 1.08, 1.12 | **<0.001** | 1.10 | 1.08, 1.12 | **<0.001** |
| **Sex** |  |  |  |  |  |  |  |  |  |  |  |  |  |  |  |  |  |  |  |  |  |  |  |  |  |  |  |
| Female | Ref | Ref | Ref | Ref | Ref | Ref | Ref | Ref | Ref | Ref | Ref | Ref | Ref | Ref | Ref | Ref | Ref | Ref | Ref | Ref | Ref | Ref | Ref | Ref | Ref | Ref | Ref |
| Male | 1.15 | 0.89, 1.49 | 0.3 | 1.15 | 0.88, 1.49 | 0.30 | 1.16 | 0.89, 1.50 | 0.3 | 1.16 | 0.89, 1.50 | 0.30 | 1.15 | 0.88, 1.51 | 0.3 | 1.16 | 0.89, 1.51 | 0.3 | 1.30 | 0.92, 1.85 | 0.13 | 1.35 | 0.95, 1.92 | 0.091 | 1.35 | 0.95, 1.92 | 0.092 |
| **Education** |  |  |  |  |  |  |  |  |  |  |  |  |  |  |  |  |  |  |  |  |  |  |  |  |  |  |  |
| Above High School/GED | Ref | Ref | Ref | Ref | Ref | Ref | Ref | Ref | Ref | Ref | Ref | Ref | Ref | Ref | Ref | Ref | Ref | Ref | Ref | Ref | Ref | Ref | Ref | Ref | Ref | Ref | Ref |
| High School/GED | 1.47 | 1.08, 2.01 | **0.014** | 1.46 | 1.07, 1.98 | **0.017** | 1.44 | 1.05, 1.96 | **0.022** | 1.50 | 1.09, 2.06 | **0.012** | 1.47 | 1.07, 2.03 | **0.017** | 1.46 | 1.06, 2.01 | **0.019** | 2.14 | 1.15, 3.98 | **0.016** | 2.27 | 1.22, 4.23 | **0.010** | 2.25 | 1.21, 4.21 | **0.011** |
| Less than High School/GED | 3.00 | 2.09, 4.31 | **<0.001** | 2.88 | 2.00, 4.14 | **<0.001** | 2.91 | 2.02, 4.19 | **<0.001** | 3.11 | 2.15, 4.49 | **<0.001** | 2.95 | 2.04, 4.27 | **<0.001** | 2.97 | 2.05, 4.30 | **<0.001** | 5.28 | 2.81, 9.91 | **<0.001** | 5.67 | 3.00, 10.7 | **<0.001** | 5.66 | 2.99, 10.7 | **<0.001** |
| **Poverty Status** |  |  |  |  |  |  |  |  |  |  |  |  |  |  |  |  |  |  |  |  |  |  |  |  |  |  |  |
| Above Poverty threshold | Ref | Ref | Ref | Ref | Ref | Ref | Ref | Ref | Ref | Ref | Ref | Ref | Ref | Ref | Ref | Ref | Ref | Ref | Ref | Ref | Ref | Ref | Ref | Ref | Ref | Ref | Ref |
| Below Poverty threshold | 1.46 | 1.08, 1.99 | **0.014** | 1.47 | 1.08, 1.99 | **0.014** | 1.47 | 1.08, 2.00 | **0.014** | 1.47 | 1.08, 1.99 | **0.015** | 1.46 | 1.07, 1.99 | **0.017** | 1.46 | 1.07, 1.99 | **0.016** | 1.55 | 1.05, 2.26 | **0.026** | 1.51 | 1.03, 2.22 | **0.034** | 1.51 | 1.03, 2.22 | **0.036** |
| **APOE E4 status** |  |  |  |  |  |  |  |  |  |  |  |  |  |  |  |  |  |  |  |  |  |  |  |  |  |  |  |
| No copies of e4 | Ref | Ref | Ref | Ref | Ref | Ref | Ref | Ref | Ref | Ref | Ref | Ref | Ref | Ref | Ref | Ref | Ref | Ref | Ref | Ref | Ref | Ref | Ref | Ref | Ref | Ref | Ref |
| Any copies of e4 | 0.91 | 0.71, 1.16 | 0.4 | 0.91 | 0.71, 1.16 | 0.40 | 0.90 | 0.70, 1.15 | 0.4 | 0.89 | 0.69, 1.14 | 0.40 | 0.89 | 0.69, 1.14 | 0.4 | 0.88 | 0.69, 1.14 | 0.3 | 1.41 | 1.02, 1.94 | **0.039** | 1.43 | 1.03, 1.98 | **0.031** | 1.42 | 1.03, 1.97 | **0.034** |
| **Social Ladder** | 0.99 | 0.92, 1.05 | 0.7 | 0.97 | 0.91, 1.04 | 0.50 | 0.98 | 0.91, 1.04 | 0.5 | 0.99 | 0.93, 1.06 | 0.80 | 0.98 | 0.92, 1.05 | 0.6 | 0.98 | 0.92, 1.05 | 0.6 | 1.09 | 1.00, 1.19 | **0.048** | 1.09 | 1.00, 1.19 | **0.049** | 1.09 | 1.00, 1.19 | **0.048** |
| **Baseline wave** |  |  |  |  |  |  |  |  |  |  |  |  |  |  |  |  |  |  |  |  |  |  |  |  |  |  |  |
| Wave 1 (2008) | Ref | Ref | Ref | Ref | Ref | Ref | Ref | Ref | Ref | Ref | Ref | Ref | Ref | Ref | Ref | Ref | Ref | Ref | Ref | Ref | Ref | Ref | Ref | Ref | Ref | Ref | Ref |
| Wave 2 (2010) | 1.15 | 0.90, 1.47 | 0.3 | 1.13 | 0.88, 1.45 | 0.30 | 1.12 | 0.87, 1.43 | 0.4 | 1.16 | 0.91, 1.49 | 0.20 | 1.14 | 0.89, 1.47 | 0.3 | 1.13 | 0.88, 1.45 | 0.3 | 1.08 | 0.78, 1.49 | 0.6 | 1.06 | 0.77, 1.47 | 0.7 | 1.06 | 0.77, 1.47 | 0.7 |
| **PGS-AD** |  |  |  |  |  |  | 1.11 | 0.94, 1.31 | 0.2 |  |  |  |  |  |  | 1.11 | 0.94, 1.31 | 0.2 |  |  |  |  |  |  |  |  |  |
| Below 75% | - | - | - | Ref | Ref | Ref | Ref | Ref | Ref | - | - | - | Ref | Ref | Ref | Ref | Ref | Ref | - | - | - | Ref | Ref | Ref | Ref | Ref | Ref |
| Above 75% | - | - | - | 1.14 | 0.86, 1.52 | 0.40 | 1.49 | 0.97, 2.29 | 0.072 | - | - | - | 1.11 | 0.83, 1.49 | 0.5 | 1.38 | 0.89, 2.14 | 0.2 | - | - | - | 1.03 | 0.68, 1.57 | 0.9 | 1.11 | 0.60, 2.05 | 0.7 |
| **Neighborhood* PGS-AD** | - | - | - | - | - | - | 1.25 | 0.89, 1.76 | 0.20 | - | - | - | - | - | - | 0.71 | 0.42, 1.22 | 0.2 | - | - | - | - | - | - | 0.89 | 0.41, 1.92 | 0.8 |
| RERI: The most disadvantaged neighborhoods*PGS-AD Above 75% | - | - | - | - | - | - | -0.19 | -0.55, 0.17 |  | - | - | - | - | - | - | -0.19 | -0.54, 0.16 |  | - | - | - | - | - | - | -0.52 | -1.28, 0.24 |  |
| **Smoking status** |  |  |  |  |  |  |  |  |  |  |  |  |  |  |  |  |  |  |  |  |  |  |  |  |  |  |  |
| Never Smoker | Ref | Ref | Ref | Ref | Ref | Ref | Ref | Ref | Ref | Ref | Ref | Ref | Ref | Ref | Ref | Ref | Ref | Ref | Ref | Ref | Ref | Ref | Ref | Ref | Ref | Ref | Ref |
| Current Smoker | 1.24 | 0.88, 1.74 | 0.2 | 1.28 | 0.91, 1.79 | 0.20 | 1.25 | 0.89, 1.76 | 0.2 | 1.27 | 0.90, 1.79 | 0.20 | 1.31 | 0.93, 1.84 | 0.13 | 1.28 | 0.91, 1.81 | 0.2 | 1.34 | 0.80, 2.27 | 0.3 | 1.38 | 0.82, 2.34 | 0.2 | 1.38 | 0.82, 2.35 | 0.2 |
| Former Smoke | 0.98 | 0.75, 1.28 | >0.9 | 1.03 | 0.79, 1.36 | 0.80 | 1.02 | 0.78, 1.34 | 0.9 | 0.96 | 0.73, 1.25 | 0.70 | 0.99 | 0.76, 1.31 | >0.9 | 0.98 | 0.75, 1.30 | >0.9 | 1.45 | 1.02, 2.07 | **0.040** | 1.48 | 1.04, 2.12 | **0.032** | 1.48 | 1.03, 2.11 | **0.034** |
| **Drinking (# drinks/day when drinks)** | 1.04 | 0.95, 1.14 | 0.4 | 1.04 | 0.95, 1.14 | 0.40 | 1.04 | 0.95, 1.14 | 0.4 | 1.04 | 0.95, 1.15 | 0.40 | 1.04 | 0.95, 1.15 | 0.4 | 1.05 | 0.95, 1.15 | 0.3 | 1.00 | 0.87, 1.15 | >0.9 | 0.99 | 0.86, 1.14 | 0.9 | 0.99 | 0.86, 1.14 | 0.9 |
| **Depression** | 1.07 | 1.00, 1.13 | **0.041** | 1.06 | 1.00, 1.13 | 0.057 | 1.06 | 1.00, 1.13 | 0.063 | 1.07 | 1.01, 1.14 | **0.031** | 1.07 | 1.00, 1.14 | **0.043** | 1.07 | 1.00, 1.14 | **0.049** | 1.12 | 1.04, 1.21 | **0.004** | 1.12 | 1.04, 1.22 | **0.003** | 1.12 | 1.04, 1.22 | **0.003** |
| **BMI** | 0.99 | 0.97, 1.01 | 0.4 | 0.99 | 0.97, 1.01 | 0.40 | 0.99 | 0.97, 1.01 | 0.4 | 0.99 | 0.98, 1.01 | 0.50 | 0.99 | 0.98, 1.01 | 0.5 | 0.99 | 0.98, 1.01 | 0.5 | 0.99 | 0.96, 1.02 | 0.5 | 0.99 | 0.96, 1.01 | 0.4 | 0.99 | 0.96, 1.01 | 0.4 |
| **Ever have Diabetes** |  |  |  |  |  |  |  |  |  |  |  |  |  |  |  |  |  |  |  |  |  |  |  |  |  |  |  |
| No | Ref | Ref | Ref | Ref | Ref | Ref | Ref | Ref | Ref | Ref | Ref | Ref | Ref | Ref | Ref | Ref | Ref | Ref | Ref | Ref | Ref | Ref | Ref | Ref | Ref | Ref | Ref |
| Yes | 1.14 | 0.83, 1.55 | 0.400 | 1.18 | 0.86, 1.61 | 0.30 | 1.17 | 0.86, 1.61 | 0.3 | 1.15 | 0.84, 1.58 | 0.40 | 1.18 | 0.86, 1.63 | 0.3 | 1.18 | 0.86, 1.63 | 0.3 | 0.92 | 0.60, 1.41 | 0.7 | 0.99 | 0.64, 1.51 | >0.9 | 0.98 | 0.64, 1.51 | >0.9 |
| **Brain Condition** |  |  |  |  |  |  |  |  |  |  |  |  |  |  |  |  |  |  |  |  |  |  |  |  |  |  |  |
| No | Ref | Ref | Ref | Ref | Ref | Ref | Ref | Ref | Ref | Ref | Ref | Ref | Ref | Ref | Ref | Ref | Ref | Ref | Ref | Ref | Ref | Ref | Ref | Ref | Ref | Ref | Ref |
| Yes | 1.32 | 0.98, 1.79 | 0.069 | 1.37 | 1.01, 1.86 | **0.042** | 1.38 | 1.01, 1.86 | **0.040** | 1.32 | 0.97, 1.79 | 0.078 | 1.36 | 1.00, 1.86 | 0.050 | 1.37 | 1.00, 1.86 | 0.048 | 1.00 | 0.65, 1.54 | >0.9 | 0.97 | 0.63, 1.49 | 0.9 | 0.96 | 0.63, 1.48 | 0.9 |
| **Chronic Condition** |  |  |  |  |  |  |  |  |  |  |  |  |  |  |  |  |  |  |  |  |  |  |  |  |  |  |  |
| None | Ref | Ref | Ref | Ref | Ref | Ref | Ref | Ref | Ref | Ref | Ref | Ref | Ref | Ref | Ref | Ref | Ref | Ref | Ref | Ref | Ref | Ref | Ref | Ref | Ref | Ref | Ref |
| 1 - 2 | 0.61 | 0.43, 0.87 | **0.006** | 0.61 | 0.43, 0.88 | **0.008** | 0.62 | 0.43, 0.88 | **0.008** | 0.59 | 0.41, 0.84 | **0.003** | 0.59 | 0.41, 0.85 | **0.004** | 0.59 | 0.41, 0.85 | **0.005** | 0.80 | 0.47, 1.34 | 0.4 | 0.80 | 0.47, 1.34 | 0.4 | 0.79 | 0.47, 1.34 | 0.4 |
| >= 3 | 0.81 | 0.53, 1.25 | 0.3 | 0.80 | 0.52, 1.24 | 0.30 | 0.81 | 0.52, 1.26 | 0.40 | 0.80 | 0.51, 1.23 | 0.30 | 0.79 | 0.51, 1.24 | 0.3 | 0.81 | 0.52, 1.26 | 0.3 | 0.68 | 0.37, 1.26 | 0.2 | 0.64 | 0.35, 1.20 | 0.2 | 0.65 | 0.35, 1.22 | 0.2 |
| **Eyesight** |  |  |  |  |  |  |  |  |  |  |  |  |  |  |  |  |  |  |  |  |  |  |  |  |  |  |  |
| Excellent | Ref | Ref | Ref | Ref | Ref | Ref | Ref | Ref | Ref | Ref | Ref | Ref | Ref | Ref | Ref | Ref | Ref | Ref | Ref | Ref | Ref | Ref | Ref | Ref | Ref | Ref | Ref |
| Very Good | 0.88 | 0.49, 1.60 | 0.7 | 0.87 | 0.47, 1.58 | 0.60 | 1.14 | 0.62, 2.07 | 0.70 | 0.85 | 0.47, 1.55 | 0.6 | 0.84 | 0.46, 1.55 | 0.6 | 0.82 | 0.44, 1.50 | 0.5 | 0.65 | 0.24, 1.79 | 0.4 | 0.58 | 0.21, 1.60 | 0.3 | 0.58 | 0.21, 1.59 | 0.3 |
| Good | 0.95 | 0.54, 1.65 | 0.9 | 0.94 | 0.54, 1.64 | 0.80 | 0.92 | 0.53, 1.61 | 0.80 | 0.94 | 0.54, 1.64 | 0.8 | 0.94 | 0.53, 1.64 | 0.8 | 0.92 | 0.53, 1.61 | 0.8 | 0.73 | 0.28, 1.88 | 0.5 | 0.64 | 0.24, 1.66 | 0.4 | 0.63 | 0.24, 1.65 | 0.4 |
| Fair | 1.12 | 0.61, 2.03 | 0.7 | 1.14 | 0.62, 2.07 | 0.70 | 1.27 | 0.63, 2.55 | 0.50 | 1.11 | 0.61, 2.03 | 0.7 | 1.13 | 0.62, 2.07 | 0.7 | 1.13 | 0.62, 2.08 | 0.7 | 1.08 | 0.41, 2.85 | 0.9 | 1.03 | 0.39, 2.72 | >0.9 | 1.03 | 0.39, 2.72 | >0.9 |
| Poor | 1.18 | 0.59, 2.36 | 0.6 | 1.28 | 0.64, 2.57 | 0.50 | 0.83 | 0.45, 1.52 | 0.50 | 1.13 | 0.56, 2.27 | 0.7 | 1.23 | 0.61, 2.47 | 0.6 | 1.22 | 0.60, 2.46 | 0.6 | 1.46 | 0.53, 4.08 | 0.5 | 1.37 | 0.49, 3.82 | 0.5 | 1.36 | 0.49, 3.80 | 0.6 |
| **Hearing** |  |  |  |  |  |  |  |  |  |  |  |  |  |  |  |  |  |  |  |  |  |  |  |  |  |  |  |
| Excellent | Ref | Ref | Ref | Ref | Ref | Ref | Ref | Ref | Ref | Ref | Ref | Ref | Ref | Ref | Ref | Ref | Ref | Ref | Ref | Ref | Ref | Ref | Ref | Ref | Ref | Ref | Ref |
| Very Good | 0.81 | 0.57, 1.14 | 0.2 | 0.83 | 0.58, 1.17 | 0.30 | 0.54 | 0.33, 0.87 | 0.012 | 0.78 | 0.55, 1.10 | 0.2 | 0.79 | 0.56, 1.12 | 0.2 | 0.80 | 0.56, 1.13 | 0.2 | 0.86 | 0.51, 1.44 | 0.6 | 0.85 | 0.51, 1.43 | 0.5 | 0.85 | 0.51, 1.44 | 0.6 |
| Good | 1.07 | 0.78, 1.47 | 0.7 | 1.06 | 0.77, 1.45 | 0.70 | 1.06 | 0.77, 1.45 | 0.70 | 1.05 | 0.76, 1.44 | 0.8 | 1.04 | 0.76, 1.44 | 0.8 | 1.04 | 0.76, 1.43 | 0.8 | 1.04 | 0.66, 1.64 | 0.9 | 1.03 | 0.65, 1.63 | 0.9 | 1.03 | 0.65, 1.63 | >0.9 |
| Fair | 0.55 | 0.34, 0.89 | **0.014** | 0.54 | 0.33, 0.87 | **0.012** | 0.81 | 0.36, 1.83 | 0.60 | 0.52 | 0.32, 0.84 | **0.008** | 0.51 | 0.31, 0.83 | **0.007** | 0.51 | 0.31, 0.83 | **0.007** | 0.92 | 0.52, 1.61 | 0.8 | 0.95 | 0.53, 1.67 | 0.8 | 0.94 | 0.53, 1.67 | 0.8 |
| Poor | 0.78 | 0.35, 1.76 | 0.6 | 0.80 | 0.35, 1.81 | 0.60 | 0.84 | 0.59, 1.18 | 0.30 | 0.73 | 0.32, 1.66 | 0.5 | 0.76 | 0.33, 1.74 | 0.5 | 0.77 | 0.34, 1.76 | 0.5 | 0.64 | 0.19, 2.15 | 0.5 | 0.62 | 0.19, 2.09 | 0.4 | 0.63 | 0.19, 2.11 | 0.5 |

Each model further adjusted for Smoking, Alcohol Consumption, BMI, Diabetes, Depression, Brian Condition (stroke, psychiatric problems), Chronic Condition (high blood pressure, diabetes, cancer, lung disease, heart disease, and arthritis), Eyesight, and Hearing.
