# Supplementary material for: Associations of perceived neighborhood factors and Alzheimer’s disease polygenic score with cognition: Evidence from the Health and Retirement Study
Source: PLoS One. 2025 Nov 20;20(11):e0336403. doi: 10.1371/journal.pone.0336403 (PMC12633890; doi:10.1371/journal.pone.0336403)
Supplement: Attachment — Submitted filename: Response_to_Reviewers.docx [file pone.0336403.s007.docx]

Dear editor and reviewers,

Please find here our point-by-point response to comments and suggestions. We appreciate the time you have taken to carefully read our manuscript and we have likewise taken care to address each comment and editorial request.

Erin Ware, on behalf of PONE-D-25-46504 co-authors.

We have included this rebuttal letter with responses to each point. We are including a marked-up, track changes version of our manuscript and an unmarked, clean version of our manuscript.

**Journal Requirements:**

We have addressed these additional requirements:

1. Please ensure that your manuscript meets PLOS ONE's style requirements, including those for file naming. The PLOS ONE style templates can be found at

2. Your ethics statement should only appear in the Methods section of your manuscript. If your ethics statement is written in any section besides the Methods, please delete it from any other section.

*Thank you, we have added an “Ethics” subsection to the end of the Methods section wherein the ethics statement has been moved.*

3. Please include captions for your Supporting Information files at the end of your manuscript, and update any in-text citations to match accordingly. Please see our

*We have included caption titles for all Supporting information at the end of the manuscript and updated in-text citations to match the required formatting.*

**Review Comments to the Author**
Reviewer #1: This is a rigorous and creative analytic approach. It not only focuses on the primary research topic (neighborhood environment) but also simultaneously takes dementia polygenic risk into account.

*We appreciate the summary comments from the reviewer on our rigorous and appropriate statistical analysis of the Health and Retirement Study data we present in this manuscript.  We address their questions below.*

I only have a few questions to raise:

**R1.1:** In the covariates included for the sensitivity analysis, according to The Lancet 2024 dementia risk factor report, the following are missing: hearing impairment, visual impairment, traumatic brain injury, and air pollution.
(I understand there may be design limitations, especially in measuring air pollution, but please at least acknowledge this in the limitations section.)

In addition to the above, I personally believe that substance use and epilepsy should also be considered.

*We agree with the reviewer and attempt to use the Lancet 2024 identified dementia risk factors wherever possible in our work. Currently within the HRS sample, we have a self-reported eyesight and hearing status rating (excellent, very good, good, fair, poor, [blind]). We have added these two variables to our sensitivity analysis* ***(revised S4 Table****) and a description of the variables in the main methods text. This includes updates to* ***Table 1*** *and* ***S1, S2,*** *and* ***S6 Tables****.*

*Per air pollution and traumatic brain injury, these variables are not available for analysis (air pollution) or not reliable measures (concussion/head injury prior to age 16) of the construct. We have therefore left them out of the analyses. We have included both smoking and alcohol use in our health behavior variable set as recommended by the Lancet 2024 report. The HRS does ask respondents at their first interview when enrolled in the study if they had any of a set of childhood diseases, including epilepsy or seizures before age 16. However, the prevalence of epilepsy or seizures is too small to include as a covariate in our sample and estimates would make our models unstable (e.g. in the 2008 core interview, 85 out of 12275 respondents or 0.7% responded ‘yes.’).*

**R1.2:** Regarding the categorization of chronic conditions (0, 1–2, ≥3), I think this approach is debatable. I recommend that brain-related conditions (psychiatric disorders, stroke) be separated out, while the remaining physical comorbidities could either be assessed using the CCI score or treated as individual covariates.

Per this reviewer’s recommendation, we have split the chronic conditions variable into ‘brain-related conditions’ and ‘other chronic conditions.’ We have categorized ‘brain-related conditions’ as either ‘yes’ or ‘no’ to whether the respondent reported a history of psychiatric disorders or stroke (in our sample, none of the respondents reported both). The remaining comorbidities were categorized as none (reference), 1-2, or greater than/equal to 3. We have included these new variables in our revised **S4 Table** and described the coding in the methods section.

Reviewer #2: This cohort study in HRS (2008–2020) examines whether perceived neighborhood disadvantage and Alzheimer’s disease polygenic score (APOE-excluded) predict incident cognitive impairment. In European-ancestry participants, both exposures independently associate with higher risk; no multiplicative or additive interaction is detected. African-ancestry estimates are imprecise. I only have a few comments on this article.

We very much thank the reviewer for taking the time to carefully read our manuscript and for their thoughtful comments and suggestions.

**R2.1:** Abstract
1. In the Results, please avoid vague phrases such as “similar effect sizes” and “comparable but nonsignificant trends were noted in the African-ancestry sample”; instead, report the numerical estimates (e.g., HRs with 95% CIs).

Thank you for this helpful suggestion. We have updated the abstract to include HRs with 95%CIs as well as the ambiguous ‘similar effect sizes’ statement to be more concise.

**R2.2:** Results
2. Given the small African ancestries sample and limited power, the phrasing “comparable but nonsignificant trends” should be tempered to “evidence was inconclusive in African ancestries due to limited precision.”

We have updated our language around the African ancestries results to reflect the limited power in the sample. This was done in both the abstract (line 43) and throughout the manuscript.

**R2.3:** Discussion
3. Replace any phrasing implying the absence of interaction with language that emphasizes evidence not detected and limited precision, especially for African ancestries: e.g., “We found no evidence of multiplicative or additive interaction; however, power to detect small-to-moderate effects—particularly in African ancestries—was limited.”

We appreciate the recommendations to temper our conclusions with a note about limited precision and power, particularly in the African ancestries sample. We have updated our results section on line 389-393 as well as lines 399-400 as well as in the abstract line 44. We have also updated our discussion with language reflecting the recommendations of this reviewer in the paragraph beginning on line 423 of the discussion.

**R2.4:** 4. Avoid “comparable but nonsignificant trends.” Use: “Estimates in African ancestries were directionally similar but imprecise, and the evidence was inconclusive due to limited precision and cross-ancestry PRS transferability.”

Thank you for this recommendation. We have updated our abstract, discussion and mentions of “comparable but nonsignificant trends” throughout the manuscript to better represent the methodological and sample challenges noted by this reviewer.
